# Recent Advances in Nanomedicine-Enhanced Photodynamic Therapy in Cutaneous Oncology: Melanoma-Dominant Evidence for Immunogenic Cell Death and Photoimmunotherapy

**DOI:** 10.3390/ijms27198723

**Published:** 2026-09-29

**Authors:** Dorota Bartusik-Aebisher, Izabella Wilk, David Aebisher

**Affiliations:** 1 Department of Biochemistry and General Chemistry, Medical College, Rzeszów University, 35-310 Rzeszów, Poland; dbartusikaebisher@ur.edu.pl; 2 English Division Science Club, Medical College, Rzeszów University, 35-310 Rzeszów, Poland; iw135750@stud.ur.edu.pl; 3 Department of Photomedicine and Physical Chemistry, Medical College, Rzeszów University, 35-310 Rzeszów, Poland

**Keywords:** photodynamic therapy, nanomedicine, skin cancer, immunogenic cell death, photoimmunotherapy, tumour microenvironment

## Abstract

Although photodynamic therapy (PDT) is a recognised treatment for the premalignant keratinocytic lesion actinic keratosis (AK) and selected superficial skin cancers, poor photosensitiser delivery, restricted light penetration and tumour hypoxia can significantly restrict its effectiveness. Recent advances in nanomedicine are extending PDT past improved transport of the photosensitiser. These approaches can also control tumour accumulation, intracellular localisation and the generation of reactive oxygen species. The degree of tumour cell damage is not the only outcome that can be influenced by these features. They can also influence tumour-cell death and immunogenic cell death (ICD)-associated danger signalling and, in selected preclinical systems, support antigen presentation and T-cell activation. Nanomedicine-enhanced PDT has increasingly been integrated with immune-checkpoint blockade and immune adjuvants in melanoma models. In selected preclinical melanoma models, these combinations have improved local tumour control and produced antitumour effects at untreated distant or metastatic sites; functional immune memory has been demonstrated only in a smaller subset of studies using tumour-rechallenge models. Nanotechnology may further address the practical limitations of conventional PDT while improving treatment precision. This is shown by skin-directed delivery, oxygen-generating systems and theranostic platforms. However, immune-related evidence remains predominantly preclinical and melanoma-based, whereas human nanomedicine evidence in AK and basal cell carcinoma (BCC) largely concerns nanoformulation-assisted PDT rather than immune modulation. Overall, the field is progressing from nanoparticle-assisted phototoxicity towards integrated photoimmunotherapy. Treatment delivery, tumour destruction and immune activation are increasingly being considered together. Together, these findings support the continued development of nanomedicine-enhanced PDT across cutaneous oncology, while emphasising that the most advanced immune and photoimmunotherapy evidence currently remains melanoma-based.

## 1. Introduction

The cutaneous disease spectrum relevant to this review includes malignant skin tumours and premalignant lesions associated predominantly with chronic ultraviolet (UV) radiation-induced damage. Actinic keratosis (AK) is a common premalignant keratinocytic lesion that occurs on chronically sun-exposed skin and is a potential precursor of cutaneous squamous cell carcinoma (cSCC) [1]. Basal cell carcinoma (BCC) and cSCC are keratinocyte malignancies, whereas melanoma is a distinct melanocytic malignancy. BCC is the most common skin malignancy and usually develops slowly, although delayed treatment may result in substantial local tissue destruction and disfigurement [2]. Local treatment is usually also sufficient to manage cSCC. However, there is a clinically relevant subset that is associated with the development of recurrence, metastasis or disease-related mortality [3]. In Australia, the persons-based incidence of keratinocyte cancer excisions was estimated at 1531 per 100,000 persons-years from 2011–2014 [4]. A less common but significantly more aggressive form of cancer is melanoma. This type of cancer has a strong capacity for metastatic dissemination [5]. In Australia, the estimated age-standardised incidence rates of invasive melanoma in 2021 were 67.8 cases per 100,000 males, and 45.4 cases per 100,000 females [6]. Most localised skin cancers are still mainly treated by surgery. However, healthy tissue may be removed by repeated or extensive excision. This can result in functional or cosmetic morbidity. Therefore, effective and minimally invasive therapies are very useful for the treatment of these superficial lesions and field cancerization.

Three components are required for photodynamic therapy (PDT). These include a photosensitiser, light at a wavelength that is absorbed by that photosensitiser, and molecular oxygen [7]. After light activation, the surrounding molecules receive energy or electrons from the excited photosensitiser. This results in the generation of singlet oxygen and other reactive oxygen species (ROS). Cellular membranes, proteins, nucleic acids and tumour vasculature are damaged by these ROS [7]. Cytotoxicity is mostly observed in tissue that contains the photosensitiser and has been exposed to light, in the presence of molecular oxygen. PDT is therefore known to provide spatially controlled local treatment. The use of PDT in dermatology is particularly well established for the treatment of AK and superficial BCC, as well as squamous cell carcinoma in situ. Multiple lesions or field cancerization can be treated with PDT, and positive cosmetic outcomes are offered by its use [8,9].

Even though conventional PDT has many advantages, we should also consider some important physicochemical and biological limitations. Hydrophobicity, poor solubility in aqueous media and a tendency for aggregation can be seen with the use of many photosensitisers. This can in turn reduce the absorption of light and ROS generation [10,11]. The uptake of these photosensitisers may also be inadequate or non-selective. This may limit their intratumoral concentration and lead to increased off-target photosensitivity [10]. Additionally, there is a limited depth to which visible light can penetrate tissue, and oxygen-dependent ROS formation can be limited by tumour hypoxia. Tumour hypoxia may progress as oxygen is consumed during irradiation [12,13]. PDT can stimulate inflammatory signalling and tumour-cell death accompanied by immunogenic cell death (ICD)-associated danger signalling; however, whether these responses translate into effective antitumour immunity varies. An immunosuppressive tumour microenvironment (TME) may significantly limit this response [13,14].

This review therefore aims to evaluate recent nanomedicine approaches which have been designed to improve photosensitiser solubility, stability and tumour delivery; enhance ROS generation; and address hypoxia and light-related limitations, whilst promoting ICD-associated signalling and, where functionally demonstrated, antitumour immune responses [15]. Special attention is paid to nanosystems that regulate the TME and allow for the development of photoimmunotherapy combinations. This includes the co-delivery of photosensitisers with immune stimulants or checkpoint inhibitors [15,16]. Importantly, the recent evidence is unevenly distributed across cutaneous disease types. Advanced mechanistic and immune-focused nano-PDT studies are concentrated predominantly in melanoma models, whereas PDT is clinically established mainly for AK, superficial BCC and cSCC in situ. Melanoma-derived findings are therefore not assumed to apply to other cutaneous lesions without direct supporting evidence.

## 2. Methods

### 2.1. Literature Search

This work was conducted as a critical narrative review of recent developments in nanomedicine-enhanced photodynamic therapy (PDT) in cutaneous oncology. PubMed was used as the primary bibliographic database, and PubMed Central (PMC) was searched as a complementary full-text resource. Although substantial overlap exists between the two resources, PMC was additionally searched to facilitate identification of relevant mechanistic and therapeutic evidence within the full text of articles. Publications identified through both resources were treated as the same publication rather than as independent evidence.

Literature searches were conducted from June to August 2026 and were updated on 20 September 2026. Search concepts covered nanomedicine-enhanced PDT in skin cancer and individual cutaneous disease types, including melanoma, cutaneous squamous cell carcinoma (cSCC), basal cell carcinoma (BCC) and actinic keratosis (AK), together with the major mechanistic and therapeutic themes addressed in this review. Targeted searches included immunogenic cell death (ICD), photoimmunotherapy, tumour hypoxia, microneedle-mediated delivery, organelle targeting, theranostics and the tumour microenvironment. Search terms were entered as combinations of these concepts, with the expanded database search strings incorporating Boolean operators, Medical Subject Headings (MeSH) terms and All Fields terms where applicable. The database-specific search concepts and exact expanded PubMed and PMC search strings are provided in Supplementary Table S1.

No publication-date filter was applied at the database-search stage. For the contemporary evidence synthesis, original studies published from 2023 to the final search date were prioritised, with particular emphasis on studies published in 2025–2026. Earlier original studies, clinical trials, guidelines and reviews were included where necessary to provide established or otherwise directly relevant disease-specific evidence, describe recognised PDT applications and foundational photochemical or immunological mechanisms, or provide relevant clinical and methodological context.

### 2.2. Eligibility and Study Selection

Studies were considered when they investigated nanomedicine-enhanced PDT in relevant cutaneous disease models or mechanisms directly relevant to its therapeutic development. Eligible evidence included in vitro studies, three-dimensional tumour models, animal studies and human clinical investigations. Cutaneous disease-specific evidence was prioritised, while studies from other tumour types were included selectively when they addressed emerging mechanisms for which cutaneous evidence was limited. Where noncutaneous tumour models are cited, they are identified explicitly and are used only as mechanistic or comparative evidence rather than as direct evidence for melanoma, cSCC or BCC. Where necessary to describe established PDT applications, fundamental mechanisms or the clinical context of conventional PDT, earlier studies, clinical trials, guidelines and reviews were included.

Search results were screened for relevance by one reviewer on the basis of titles and abstracts, followed by examination of the full texts of potentially relevant publications; uncertain eligibility decisions were discussed with the other authors. Studies were retained when they directly addressed nanomedicine-enhanced PDT in cutaneous oncology or provided evidence relevant to the therapeutic and mechanistic themes considered in this review. Disease-specific original studies were prioritised, whereas reviews, guidelines and earlier publications were used primarily for clinical, methodological or mechanistic context. Studies without a relevant PDT or nanomedicine component, or without direct relevance to the disease, therapeutic or mechanistic questions addressed in this review, were excluded from the central synthesis. Overlapping records identified through PubMed and PMC were treated as the same publication. No formal systematic-review screening protocol or quantitative study-selection procedure was applied.

### 2.3. Critical Evaluation and Evidence Synthesis

For the purposes of this narrative review, ‘critical evaluation’ referred to qualitative assessment of four domains: methodological quality and robustness, strength of evidence, relevance to cutaneous oncology and translational significance. Methodological robustness was considered in relation to the appropriateness of the experimental model; inclusion of relevant untreated, carrier, photosensitiser and irradiation controls; reporting of PDT conditions; suitability of the biological endpoints; and whether mechanistic conclusions were supported by functional experiments rather than surrogate markers alone.

Strength of evidence was interpreted according to experimental level, distinguishing in vitro and three-dimensional models from animal studies and human clinical investigations. Where immune effects were claimed, evidence from immunocompetent models and functional immune endpoints was considered more informative than tumour-growth effects in immunodeficient xenograft models alone. Findings supported across complementary experimental levels were considered more robust than observations confined to a single model or endpoint.

Relevance was assessed according to disease specificity and mechanistic directness. Direct evidence from melanoma, cSCC, BCC or AK was given greater relevance to cutaneous oncology than evidence derived from noncutaneous tumour models. Findings from melanoma were not assumed to apply to AK, BCC or cSCC without direct supporting evidence.

Translational significance was considered in relation to the availability of human evidence, clinical applicability of the delivery route, tumour selectivity, safety and toxicity, formulation stability, manufacturing complexity, use of appropriate comparator treatments and whether an advantage was demonstrated over the free photosensitiser or conventional PDT. For multifunctional systems, therapeutic effects attributable to additional components such as photothermal therapy, chemodynamic therapy, chemotherapy, metabolic modulation or immune-active agents were distinguished from effects attributable specifically to PDT.

Immune-related outcomes were also interpreted according to the level of functional evidence. Tumour-cell death or changes in individual damage-associated molecular patterns were regarded as evidence of ICD-associated signalling rather than definitive demonstration of immunogenicity. Dendritic-cell activation and tumour-specific T-cell responses provided stronger functional evidence, while antitumour effects at untreated distant tumours were distinguished from demonstrated systemic adaptive immunity. Protection following tumour rechallenge was considered the strongest evidence of functional antitumour immune memory among the experimental outcomes reviewed.

Findings were synthesised qualitatively according to therapeutic and mechanistic themes. As the evidence was unevenly distributed across lesion and tumour types, findings were also interpreted according to disease context and experimental level, without extrapolating melanoma-derived results to AK, BCC or cSCC in the absence of direct supporting evidence. No formal meta-analysis, numerical quality score, validated risk-of-bias assessment or systematic-review study-selection procedure was performed.

## 3. Photodynamic Therapy in Cutaneous Oncology: Mechanisms and Current Limitations

### 3.1. Current Dermatological Applications of PDT

Topical photodynamic therapy (PDT) is an established treatment for superficial lesions. This treatment commonly uses 5-aminolevulinic acid (ALA) or methyl aminolevulinate (MAL). Clinically, PDT is most strongly involved in the treatment of AK, where conventional and daylight protocols successfully achieve clearance with favourable cosmetic outcomes. In this context, PDT is also involved in the field-directed treatment of multiple lesions [17]. When the preservation of tissue or cosmetics are prioritised, selected superficial basal cell carcinomas are also treated with PDT. However, when PDT is used here instead of surgery or imiquimod, recurrence and long-term tumour free survival may be less favourable [18,19]. Another indication for treatment with topical PDT is Bowen disease, or cutaneous squamous cell carcinoma in situ. However, a randomised trial reported lower sustained clearance with MAL-PDT than with surgical excision and did not establish non-inferiority [20].

On the other hand, routine treatment for invasive cutaneous squamous cell carcinoma or melanoma does not usually involve PDT. Cell and mouse models provide most of the evidence for cSCC. The results from the studies involving these models show that tumour growth can be suppressed by ALA-PDT, and that pro-inflammatory macrophage or CD8+ T cell recruitment is also stimulated [21,22]. In melanoma, pigmentation and tumour depth can limit the delivery of light and photodynamic efficacy. For these reasons, applications remain mostly experimental. However, tumour growth in murine melanoma models has been reduced by nanoparticle-delivered photosensitisers and optical-clearing approaches [23,24]. Thus, topical PDT treatment is most clinically relevant to AK, superficial BCC and selected squamous cell carcinoma in situ.

### 3.2. Photochemical and Cellular Mechanisms

When a photosensitiser absorbs light of an appropriate wavelength, it is promoted to an excited singlet state and may subsequently undergo intersystem crossing to a longer-lived triplet state. From this state, photochemical reactions can proceed through Type I and/or Type II pathways. In Type I photochemistry, the excited photosensitiser undergoes electron or hydrogen-atom transfer with surrounding substrates, generating radical or radical-ion intermediates. Subsequent reactions can generate reactive oxygen species such as superoxide (O_2_•^−^), hydrogen peroxide (H_2_O_2_) and hydroxyl radicals (•OH). Although Type I photochemistry can be less dependent on oxygen than the Type II pathway, it is not completely oxygen-independent, because the formation of several oxygen-derived Type I ROS still requires molecular oxygen [25,26]. In Type II photochemistry, the triplet-state photosensitiser transfers energy directly to ground-state triplet molecular oxygen (^3^O_2_), producing singlet oxygen (^1^O_2_). Type I and Type II pathways can occur concurrently, and their relative contributions depend on the photosensitiser, substrate environment and oxygen availability [25,26]. Nearby membrane lipids, proteins and organelles are oxidised by the short-lived ROS that are generated in these reactions. The affected organelles include mitochondria, lysosomes and the endoplasmic reticulum [27].

The localisation of the photosensitiser and light dose that was successfully delivered have a significant influence on the response of the cell to PDT. Apoptosis is usually the result of mitochondrial injury, while necrosis is favoured following extreme damage to the membrane. Autophagy can either support stress adaptation or contribute to cell death. PDT may also trigger regulated cell-death pathways such as necroptosis, ferroptosis or pyroptosis. Importantly, ferroptosis is considered here as a lipid-peroxidation-dependent cell-death mechanism that may occur downstream of PDT or be promoted by additional nanoplatform components, rather than as a phototherapeutic modality equivalent to PDT, photothermal therapy (PTT) or chemodynamic therapy (CDT) [28]. The endothelium of tumours is damaged by PDT as well. When damaged, thrombogenic surfaces that promote platelet aggregation, vasoconstriction and an increased vascular permeability are formed [29]. Cytokines, chemokines and damage-associated molecular patterns are released because of oxidative injury and by the dying cells. These signals produce acute local inflammation and recruit neutrophils and macrophages to the site of the tumour [30].

### 3.3. Limitations of Conventional PDT

**Figure 1:**
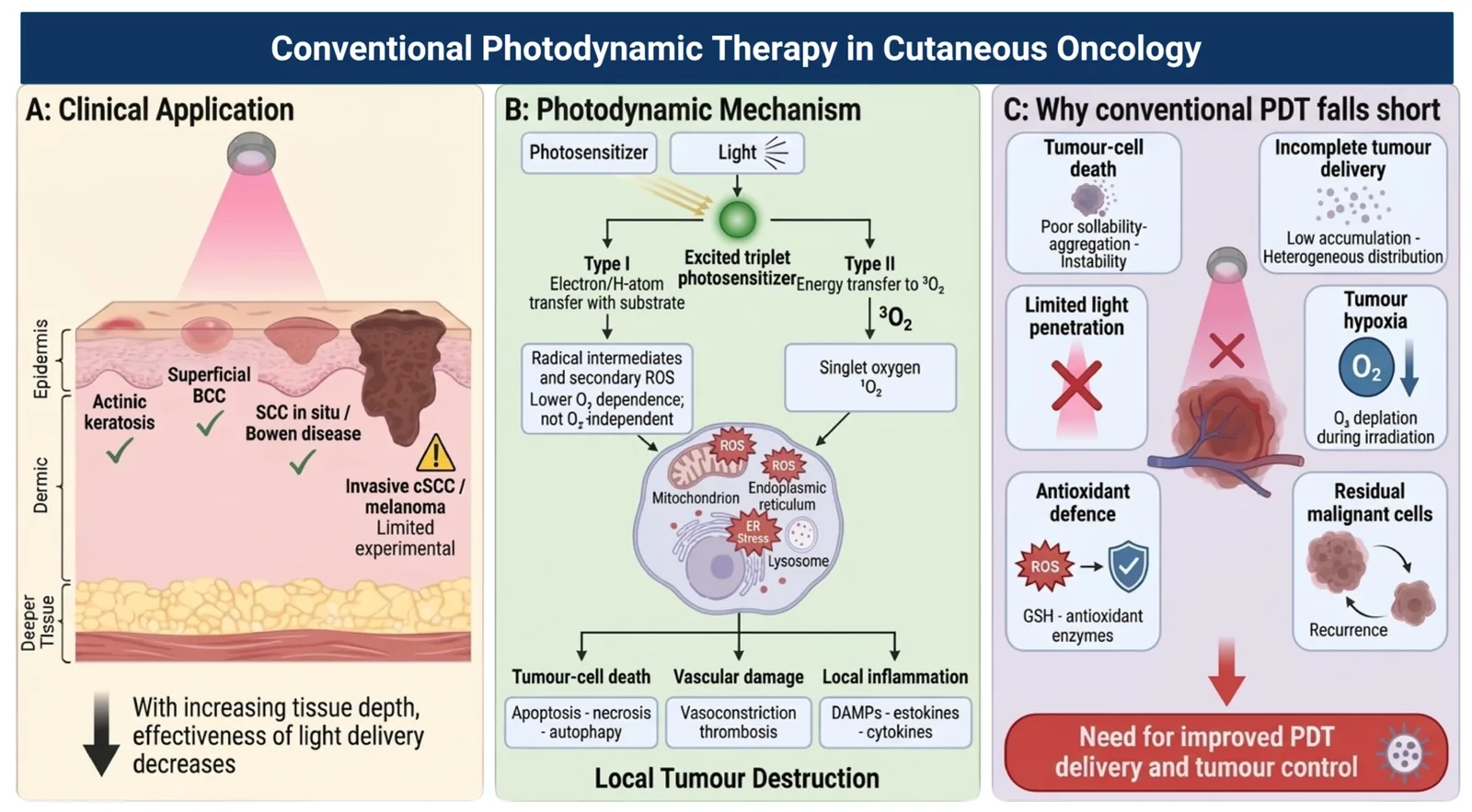
Conventional photodynamic therapy in cutaneous oncology. (**A**) Clinical applications by lesion depth. (**B**) Type I and II mechanisms and resulting tumour damage. (**C**) Key limitations, including poor delivery, limited light penetration, hypoxia and antioxidant defence. Created in BioRender. Wilk, I. (2026) https://BioRender.com/yli6ql3, accessed on 25 September 2026.

There are limitations which restrict the effectiveness of conventional PDT. Skin photosensitivity can be prolonged by photosensitiser-related limitations. These include poor aqueous solubility, aggregation, and low stability, as well as slow systemic clearance [31,32]. There are also several limitations related to the delivery of the photosensitiser to the tumour. Most significantly, these include inadequate tumour accumulation, non-specific uptake, heterogeneous intratumoral distribution and limited penetration of topical photosensitisers or activating light through skin and tumour tissue [13,33]. Pre-existing tumour hypoxia and oxygen depletion during irradiation are microenvironment-related limitations. Both of these are limitations as they reduce the generation of ROS. Oxidative stress can be neutralised by antioxidant systems, including glutathione and antioxidant enzymes. However, this can promote resistance to PDT [34,35]. Due to these factors, responses to treatment may vary between patients. In a therapeutic context, residual malignant cells may be the result of incomplete light or photosensitiser delivery. These cells may later lead to recurrence because of incomplete tumour eradication. PDT-induced antitumour immune responses can also be insufficient or variable, and the treatment of deeper lesions may be restricted by limited light penetration [13], as shown in Figure 1. These limitations provide a rationale for nanomedicine strategies which are designed to improve the delivery of photosensitisers, tumour selectivity, oxygen availability and overall therapeutic efficacy.

## 4. Nanomedicine Platforms for Enhanced Photodynamic Therapy

The nanomedicine evidence reviewed in this section is unevenly distributed across cutaneous disease types, with advanced mechanistic and multifunctional studies dominated by melanoma models. Findings from these models are therefore interpreted as melanoma-specific unless corresponding evidence is available in other cutaneous lesions or tumours.

### 4.1. Organic Nanocarriers

Organic nanocarriers can improve photosensitiser stability, skin penetration and tumour accumulation, but these formulation advantages should be distinguished from evidence of therapeutic efficacy. Recent studies range from physicochemical and in vitro assessments to animal tumour-control and immune-response experiments; therefore, enhanced phototoxicity, immunogenic-cell-death (ICD) markers and in vivo antitumour immunity should not be interpreted as equivalent outcomes. Experimental models, comparator groups, PDT conditions, quantitative outcomes and key limitations of representative studies are summarised in Table 1. The carrier classes also differ in the type and maturity of evidence currently available, which limits direct comparisons of their relative performance. Relevant biological barriers also differ between platforms, including penetration across the stratum corneum, retention within cutaneous tumours, cellular uptake, systemic or local delivery constraints and preferential accumulation in malignant rather than normal tissue. Accordingly, apparent advantages in loading capacity or phototoxicity do not necessarily translate into superior delivery or therapeutic selectivity in vivo.

Among the systems reviewed, selected liposomal, polymeric and micellar platforms provide evidence extending beyond in vitro cell viability to in vivo tumour-control experiments. Curcumin/silver-nanoparticle-loaded hybrid liposomes increased curcumin retention in skin and phototoxicity in B16-F10 melanoma cells, while intratumoral administration followed by PDT suppressed B16-F10 tumour growth and prolonged survival in mice [36]. However, the intratumoral route limits conclusions regarding systemic delivery. Similarly, ALA-loaded tea-polyphenol nanoparticles incorporated into dissolving microneedles enhanced skin penetration and ALA stability, increased protoporphyrin IX (PpIX) and ROS generation in A375 melanoma and A431 squamous-cell carcinoma cells, and inhibited both tumour models in vivo using a reduced ALA dose [37]. ER-targeted PpIX peptide micelles co-assembled with rapamycin also increased tumour accumulation and enhanced antitumour efficacy in A375 xenografts [38]. However, because rapamycin is pharmacologically active, the therapeutic effect cannot be attributed solely to enhanced PDT.

By comparison, the nanoemulsion, nanostructured lipid-carrier and protein-based systems discussed here are supported principally by in vitro therapeutic evidence. Chlorophyll-loaded castor-oil nanoemulsions produced greater PDT sensitivity in B16-F10 melanoma cells than in HaCaT keratinocytes, with IC_50_ values of approximately 313 and 545 µM, respectively [39], indicating differential cellular sensitivity. PpIX-loaded nanostructured lipid carriers similarly produced the marked light-dependent killing of A2058 melanoma cells, with no detectable dark toxicity [40]. More directly, a bovine serum albumin (BSA)–gold–phthalocyanine nanocomplex reduced A375 cell survival to 23.1%, compared with 48.7% for the non-complexed photosensitiser [41]. These studies demonstrate substantial in vitro phototoxicity, but do not establish tumour selectivity or safety in vivo. Thus, these platforms demonstrate distinct formulation or phototoxicity advantages, but the available studies do not permit a valid head-to-head assessment of their efficacy relative to the liposomal, polymeric or micellar systems evaluated in vivo.

Immunological endpoints require further distinction. AlPc-loaded solid lipid nanoparticles induced phototoxicity together with ICD-associated DAMP changes and dendritic-cell activation in B16-F10-based experiments [42]. These findings support in vitro ICD-associated DAMP signalling and dendritic-cell activation, but do not establish functional systemic antitumour immunity. In contrast, chlorin e6 (Ce6)-loaded liposomes fused with γδ-T-cell exosomes were evaluated in melanoma cells, tumour-bearing nude mice and humanised mice [43]. The hybrid vesicles accumulated 2.29-fold more strongly in A375 tumours than free Ce6 and 1.37-fold more strongly than conventional Ce6 liposomes, while also suppressing tumour growth and promoting in vivo immune activation. This contrast illustrates an important difference even within lipid-based platforms: evidence may range from in vitro ICD-associated signalling to in vivo tumour and immune responses, and these outcomes should not be treated as equivalent measures of carrier efficacy. Nevertheless, the intrinsic cytolytic and immunomodulatory activity of γδ-T-cell exosomes complicates attribution of the therapeutic effect to enhanced PDT alone.

Safety evidence should likewise not be conflated with efficacy. Rose-Bengal dendrimer formulations showed favourable short-term systemic tolerability in healthy mice [44], but the study did not include tumour-bearing animals or PDT irradiation and therefore does not establish antitumour efficacy. Formulation-stability evidence was also uneven: long-term colloidal stability was demonstrated for the chlorophyll nanoemulsion [39], whereas other systems were evaluated over substantially shorter periods or did not provide comparable stability data. Across these systems, the most consistently quantified outcomes concern in vitro phototoxicity and short-term xenograft tumour control, whereas evidence for preferential tumour selectivity, long-term toxicity and systemic antitumour immunity remains substantially less extensive. Normal-cell comparisons, long-term toxicity assessment and formulation-stability testing are also inconsistently reported. Most evidence remains preclinical and is disproportionately derived from melanoma models. Moreover, none of the organic platforms discussed here has yet established clinical efficacy for nanocarrier-enhanced PDT in skin cancer; systems supported by in vivo tumour-control data therefore represent greater experimental maturity rather than demonstrated clinical readiness. No organic carrier class therefore currently demonstrates superiority across efficacy, selectivity, safety, formulation stability and immune activity; rather, the apparent advantages are dependent on the endpoint and experimental model examined. Consequently, direct head-to-head comparisons between carrier classes and clinically relevant validation are required before the relative advantages of individual organic platforms can be established.

### 4.2. Inorganic and Hybrid Nanoplatforms

Inorganic and hybrid nanoplatforms can enhance PDT through distinct functions, including improved photosensitiser delivery, wavelength conversion, catalytic modulation of the tumour microenvironment and treatment monitoring. These mechanisms should be distinguished from photothermal therapy (PTT), in which absorbed light is converted predominantly into heat, and chemodynamic therapy (CDT), which relies on catalytic ROS generation such as Fenton or Fenton-like reactions. Therapeutic effects arising from PTT, CDT or other independently active components therefore cannot be attributed to enhanced PDT alone.

Mesoporous silica provides a relatively direct example of nanomaterial-assisted photosensitiser delivery. PpIX-loaded mesoporous silica-coated *Haliclona* sp. spicules (mSHS@PpIX) increased PpIX deposition in the viable epidermis and dermis by approximately 154-fold and 22-fold, respectively, compared with uncoated spicule-mediated delivery [45]. In B16 melanoma-bearing mice, topical mSHS@PpIX followed by 635 nm irradiation at 1 W for 15 min per session eradicated primary tumours, whereas SHS plus PpIX, topical PpIX, intravenous PpIX and irradiation-only comparator groups showed continued tumour growth; no recurrence or metastasis was reported during 60 days of follow-up. Nevertheless, this evidence remains preclinical and does not establish superiority over an established clinical PDT regimen.

Carbon dots have progressed to limited clinical evaluation. In a small uncontrolled clinical study of 22 patients with 27 BCC or Bowen-disease lesions, all lesions underwent curettage before carbon-dot PDT using 650 nm light at 100 mW/cm^2^ for 20 min (120 J/cm^2^), followed by 450 nm light at 100 mW/cm^2^ for 30 min (180 J/cm^2^) [46]. Complete responses were reported in several BCC subtypes, whereas Bowen-disease responses were less consistent; no irradiation-associated pain was reported and healing was rapid. As there was no conventional ALA- or MAL-PDT comparator and curettage was part of the treatment in every lesion, these findings provide preliminary human feasibility evidence rather than evidence of the superiority of carbon-dot PDT over established PDT. Upconversion nanoparticles provide a different function by converting near-infrared (NIR) excitation into shorter-wavelength emission capable of activating conventional photosensitisers. In A375 melanoma cells, NaYF4,Tm upconversion nanoparticles (UCNPs) combined with Rose Bengal and 980 nm irradiation at 8.3 W/cm^2^ for 5 min per cycle (2490 J/cm^2^) reduced viability to 33.5% after four cycles, whereas activated UCNPs or Rose Bengal alone maintained approximately 90% viability; indocyanine green (ICG)-PDT was also included as a photosensitiser comparator [47]. The platform additionally enabled real-time assessment of oxygen consumption and ROS dynamics, although the evidence remains entirely in vitro.

Other inorganic-hybrid systems rely on multiple therapeutic mechanisms. In A431 cutaneous squamous-cell carcinoma, hyaluronic acid (HA)-functionalised Cu-Cy@zeolitic imidazolate framework-8 (ZIF-8) combined CD44-directed targeting with singlet-oxygen generation, glutathione depletion and Fenton-like catalytic activity. Compared with CNZ/CNZH and light-only controls, CNZH reduced cell viability from 32% without irradiation to approximately 22% after 365 nm irradiation at 24 W for 10 min, with 77.1% apoptosis, and the irradiated CNZH group produced the strongest tumour suppression in xenografts [48]. As substantial light-independent CDT activity was also present, these outcomes represent synergistic CDT/PDT rather than PDT alone. Similarly, transdermal 2-deoxy-D-glucose (2-DG) deep eutectic system (DES)–mesoporous organosilicon nanoparticle (MON)@Ce6 was evaluated in melanoma cell and mouse models, where sequential 2-DG-mediated glycolytic inhibition reduced hypoxia before Ce6-mediated PDT and enhanced ICD-associated signalling and antitumour activity [49]. As 2-DG contributes independently to the response, these findings support metabolic modulation combined with PDT rather than enhanced photodynamic activity alone.

Overall, the evidence ranges from mechanistic in vitro studies to animal models and limited clinical investigation. Direct advantages over free photosensitiser are clearest where nanomaterials improve delivery or optical activation and appropriate photosensitiser controls are included, whereas catalytic and metabolically active systems should be interpreted as multimodal therapies. Long-term biodegradability, tissue persistence and clearance, repeated-dose safety and manufacturing complexity remain incompletely characterised, and clinical superiority over conventional PDT has not yet been established.

### 4.3. Tumour-Targeted and Skin-Directed Delivery

The enhanced permeability and retention effect can cause systemic nanoparticles to accumulate passively in tumours. However, this is a heterogeneous process, and it does not guarantee cellular uptake. In active targeting, nanoparticle surfaces become functionalised with ligands that recognise tumour-associated receptors [50]. In B16-F10 melanoma cells, hypericin-loaded P123 micelles functionalised with spermine and folic acid enhanced photosensitiser uptake and photoinactivation under irradiation [51]. Similarly, integrin-driven interactions with melanoma cells were enhanced by gold nanoparticles functionalised with arginine-glycine-aspartic acid (RGD) [52]. Specificity can also be improved by antibody functionalisation. ZnPcS4 bioavailability, ROS generation and phototoxicity in melanoma models were enhanced by anti-melanoma inhibitory activity (MIA) gold nanocarriers [53]. Likewise, an epidermal growth factor receptor (EGFR)-targeted affibody-indocyanine green (ICG) conjugate was seen to increase selective uptake and tumour retention in A431 skin-cancer xenografts. These xenografts were EGFR positive [54].

Systemic accumulation is not relied on for local delivery to cutaneous tumours. Within a lesion, photosensitiser residence can be increased by topical nanocarriers. With intradermal and microneedle systems, the nanoparticles can be delivered directly into viable skin. This is possible as these systems cross the stratum corneum. Hyaluronic-acid microneedle patches carrying ZnPc-containing DNA nanoflowers were developed. These patches successfully penetrated the skin barrier, relieved hypoxia and suppressed B16F10 melanoma, while simultaneously increasing antitumour activity [55]. ALA-catalase-loaded poly (lactic-co-glycolic acid) (PLGA) nanoparticles were incorporated into dissolving microneedles. These similarly enhanced transdermal delivery and the efficacy of PDT in cSCC models [56]. Local exposure can be additionally extended by hydrogels and patches. Local, tumour-responsive photoimmunotherapy of melanoma was supported by a DNA hydrogel [57]. In BCC and Bowen disease, carbon-dot PDT formulated as a topical cream or hydrogel has also undergone preliminary clinical evaluation [46]. This provides preliminary human feasibility evidence for locally delivered nanomedicine in skin cancer. These approaches may help to reduce off-target exposure and systemic photosensitivity, whilst improving local retention.

### 4.4. Stimuli-Responsive and Organelle-Targeted Nanomedicine

Stimuli-responsive nanomedicine is designed to activate or release photosensitisers in response to specific tumour-associated conditions or externally applied stimuli. In this way, the spatial control of PDT is improved. Acidic pH, enzymes and redox conditions, as well as elevated ROS are common endogenous triggers. Light and heat provide external control with better spatial precision. Recently, a pH-responsive peptide nanoplatform enhanced PDT in A375 melanoma xenografts. Under acidic conditions, it underwent structural transformation, promoting lysosomal escape and subsequent delivery of an endoplasmic reticulum (ER)-targeted photosensitiser [38]. pH-light-cascade responsiveness was used by a second melanoma system. In orthotopic melanoma, Ir-MnII/III nanoparticles dissociated in acidic lysosomes, generated oxygen and promoted light-induced ROS production; treatment was accompanied by apoptosis and ferroptosis [58]. Here, ferroptosis represents an additional cell-death mechanism contributing to the therapeutic response rather than PDT itself. In cutaneous models, enzyme-based control has also been explored. Collagenase-mediated extracellular-matrix remodelling was used in oxygenated collagenase nanoliposomes. This system enhanced PDT in deeper melanoma tissue by improving penetration and oxygen delivery [59]. Systems responsive to light and heat have been evaluated as well. Combined photodynamic, photothermal and immunotherapeutic effects against melanoma were observed in a study using NIR-activated ethosomes [60]. However, redox- and ROS-responsive nanoplatforms have been studied less extensively in skin cancer than pH- and light-responsive systems [61].

The intracellular location at which ROS are produced depends on which organelle is targeted. Respiration is disrupted by mitochondrial targeting, proteotoxic stress is promoted by ER targeting and membrane permeabilisation may be triggered by lysosomal targeting. Membrane lipids or DNA can be damaged by plasma-membrane or nuclear localisation. Recently, evidence strongest for ER, lysosomal and mitochondrial targeting has been provided by melanoma nanomedicine [38,58]. As ROS act close to where they are generated, their intracellular site of generation can have an influence on tumour killing and the release of immunogenic danger signals.

### 4.5. Oxygen-Modulating and ROS-Amplifying Strategies

PDT is limited by hypoxia, as oxygen-dependent ROS generation is reduced under this condition. The ROS that are produced can be neutralised by intracellular antioxidants. Molecular oxygen can be transported by oxygen-carrying nanoparticles, such as perfluorocarbon and haemoglobin-based systems. However, recent PDT evidence specific to skin cancer for these carriers remains limited. Recent cutaneous research has focused more strongly on in situ oxygen generation within the tumour microenvironment. An MnO_2_-containing magnetic nanocomposite converted endogenous H_2_O_2_ into oxygen, increased intracellular ROS and suppressed growth and metastasis of B16-F10 melanoma [62]. A similar approach is offered by catalase-containing nanoparticles. In cSCC models, ALA/catalase-loaded PLGA nanoparticles incorporated into dissolving microneedles relieved hypoxia and enhanced PDT [56]. Oxygen can also be generated directly within the tumour microenvironment by calcium peroxide. Oxygen was generated within the acidic melanoma microenvironment by a CaO_2_-containing core–shell microneedle platform co-delivering verteporfin and the bromodomain-containing protein 4 (BRD4)-targeting proteolysis-targeting chimera (PROTAC) ARV825. The multimodal system achieved a 90% melanoma inhibition rate and was reported to provide protection against tumour recurrence and metastasis in a preclinical melanoma model [63]. Weakening antioxidant defences can additionally result in ROS amplification. An H_2_O_2_/O_2_ self-supplying nanoplatform, evaluated in cancer cells and melanoma mouse models, depleted glutathione while increasing oxygen and oxidative stress at the same time. This enhanced oxidative stress through combined PDT/CDT; because the CDT component generates ROS catalytically rather than through photosensitiser excitation, the resulting therapeutic effect should be interpreted as multimodal rather than as enhanced PDT alone [64]. Directly inhibiting antioxidant pathways, such as by glutathione peroxidase 4 (GPX4), is another possible way to sustain lipid peroxidation. However, this approach has not been widely studied in nanomedicine-enhanced PDT for skin cancer [65]. An alternative approach is to favour Type I photochemistry, which can show lower oxygen dependence than Type II photochemistry because electron- or hydrogen-transfer reactions initiate radical pathways, although the formation of oxygen-derived Type I ROS may still require molecular oxygen [66]. Poly(9,9-dioctylfluorene-alt-benzothiadiazole) (PFBT)/1-[3-(methoxycarbonyl) propyl]-1-phenyl-[6.6]C_61_ (PCBM) heterojunction nanoparticles generated both Type I ROS, including superoxide and hydroxyl radicals, and Type II singlet oxygen, while endogenous melanoma melanin accelerated the photocatalytic cycle and further amplified ROS generation [67]. These self-supplying and self-amplifying systems directly target hypoxia and ROS resistance.

### 4.6. Theranostic Nanoplatforms

**Figure 2:**
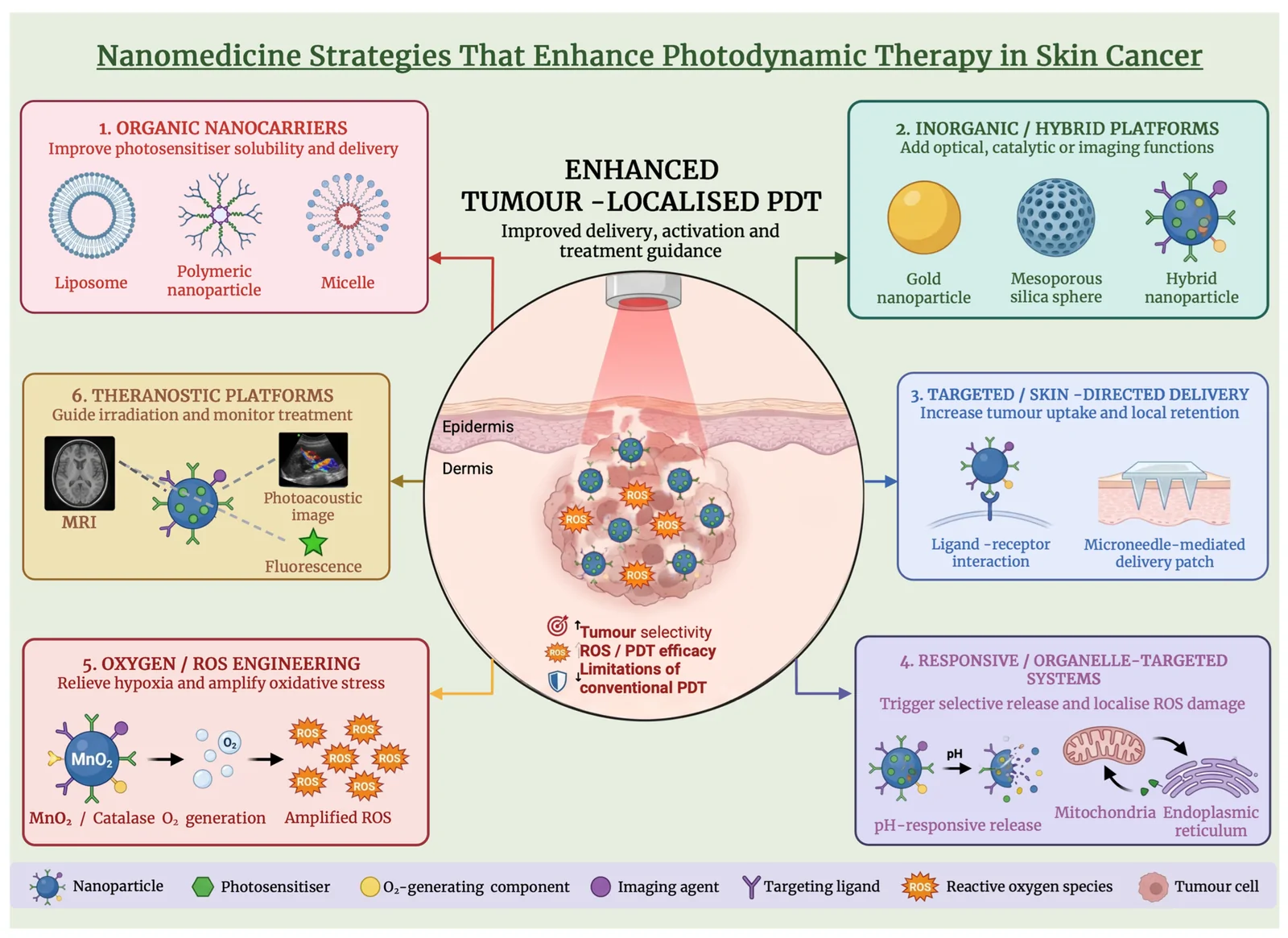
Nanomedicine strategies for enhancing photodynamic therapy in skin cancer. Nanoplatforms improve photosensitiser delivery, tumour targeting, ROS generation and treatment guidance for more effective PDT. Created in BioRender. Wilk, I. (2026) https://BioRender.com/t96q6v0, accessed on 25 September 2026.

Theranostic nanoplatforms allow photosensitiser distribution, tumour uptake and therapeutic response to be assessed within the same system, by combining treatment with imaging. As many photosensitisers or associated fluorophores emit detectable light, fluorescence imaging is quite compatible with PDT. Recent theranostic research into skin cancer successfully used near-infrared fluorophores and multifunctional nanomaterials to visualise tumour localisation. Phototherapy was also supported by this approach [68]. Greater depth is offered by photoacoustic imaging, which converts absorbed light into acoustic signals. In this type of imaging, nanoparticle accumulation, tumour vasculature and oxygenation can be mapped. However, this approach has not yet been widely evaluated in nano-PDT in skin cancer [68]. Without the use of ionising radiation, magnetic resonance imaging (MRI) can provide detailed anatomical information from deeper tissues. T2-weighted MRI in mice with B16-F10 melanoma was facilitated by superparamagnetic iron oxide (SPIO)/ICG nanomicelles. Tumour accumulation was successfully tracked with this strategy. A five-hour maximum-uptake window was identified before subsequent combined NIR PDT/PTT [69]. As both photodynamic ROS generation and photothermal heating contributed to treatment, the therapeutic response cannot be attributed specifically to PDT. Upconversion nanoparticles also allow for treatment monitoring. In an A375 melanoma study, UCNP-assisted Rose-Bengal PDT was combined with dissolved-oxygen sensing. This allowed for the assessment of oxygen consumption and ROS dynamics during irradiation in real-time [47]. When and where light should be delivered may therefore be guided by theranostic imaging. This is especially relevant because skin-cancer lesions are usually easily accessible for imaging and PDT light delivery. Both drug delivery and intradermal light distribution were shown to be improved by dissolving microneedle arrays. This system supports more spatially controlled PDT [70]. Fluorescence, photoacoustic and MRI, together with treatment-responsive sensing, can change nanoparticles from passive carriers into feedback-enabled systems, as shown in Figure 2. These systems are capable of localising lesions, guiding irradiation and monitoring if PDT is functioning properly. A summary of representative recent nanomedicine strategies used to enhance PDT in skin cancer is provided in Table 2.

## 5. Nanomedicine-Enhanced PDT and Immunogenic Cell Death

### 5.1. Definition and Molecular Features of Immunogenic Cell Death

Beyond improving direct tumour-cell killing, nanomedicine-enhanced PDT can alter the way tumour cells die, potentially converting local oxidative damage into immunogenic cell death (ICD). ICD is a type of regulated tumour-cell death that stimulates adaptive antitumour immunity, as well as removing malignant cells. ICD is different from non-immunogenic destruction, which may eliminate tumour cells without effective immune priming, as it couples cell death with tumour-antigen release and damage-associated molecular patterns (DAMPs). Antigen-presenting cells can be activated by DAMPs [75]. The uptake of tumour cells by dendritic cells is promoted by calreticulin (CRT) translocating to the cell surface and acting as an “eat-me” signal. Purinergic pathways that promote dendritic-cell (DC) recruitment and inflammatory signalling are stimulated by extracellular adenosine triphosphate (ATP), which acts as a “find-me” signal. Damaged cells release high-mobility group box 1 (HMGB1), which engages pattern-recognition receptors such as Toll-like receptor 4 (TLR4) and receptor for advanced glycation end products (RAGE). This process supports antigen processing and dendritic-cell activation [75,76]. Heat-shock protein 70 (HSP70) and heat-shock protein 90 (HSP90) can also become exposed on the surface or released extracellularly. They can then bind tumour antigens and facilitate their uptake by antigen-presenting cells [75,76].

An additional immunogenic signal is provided by tumour-derived nucleic acids. Cyclic GMP-AMP synthase (cGAS)-stimulator of interferon genes (STING) signalling can be triggered by cytosolic or extracellular DNA, including mitochondrial DNA. This leads to type I interferon production. Dendritic-cell maturation, antigen cross-presentation and tumour-specific CD8+ T-cell priming are promoted by type I interferons, particularly interferon (IFN)-α and IFN-β [75,76]. This molecular pattern is directly relevant to PDT in melanoma. CRT exposure and extracellular ATP and HMGB1 release in A375 and SK-Mel-28 melanoma cells were induced by polydopamine (PDA)-Ce6-R837 nanoparticle-mediated phototherapy. The maturation of dendritic cells was also stimulated by this approach [77]. Importantly, tumour-cell death or the detection of individual DAMPs and other immune-associated markers should be regarded as evidence of ICD-associated signalling rather than definitive proof of bona fide ICD. Demonstration of dendritic-cell maturation or tumour-specific T-cell activation provides stronger functional evidence of immunogenicity, whereas protective responses in immunocompetent syngeneic vaccination/rechallenge models provide the strongest experimental validation that the induced cell death is immunogenic [78,79].

### 5.2. From Oxidative Stress to Immune Activation

Local oxidative injury can be converted into a sequence that connects the destruction of a tumour with adaptive immunity by nanomedicine-enhanced PDT. Following the accumulation of nanoparticles within the tumour, the loaded photosensitiser is activated by light. Close to its intracellular site of localisation, ROS are generated. If the ER, mitochondria, or lysosomes are damaged by these ROS, tumour-cell death accompanied by immunogenic signalling can be promoted by organelle stress. Tumour accumulation was improved by pH-responsive peptide micelles in melanoma. This system also increased the efficacy of PDT in A375 xenografts by delivering a photosensitiser targeted at the endoplasmic reticulum [38]. A375 and SK-Mel-28 melanoma cells efficiently internalised PDA-Ce6-R837 nanoparticles. Apoptosis along with the exposure of calreticulin and the release of extracellular ATP and HMGB1 were stimulated by irradiation [77]. Dendritic cells, which engulf dying tumour cells and associated tumour antigens, are attracted and activated by these danger signals. The expression of CD80/CD86 and the production of interleukin (IL)-12p70 in dendritic cells derived from the bone marrow was increased in the same study, by nanoparticle phototherapy. This suggested enhanced maturation of dendritic cells [77].

**Figure 3:**
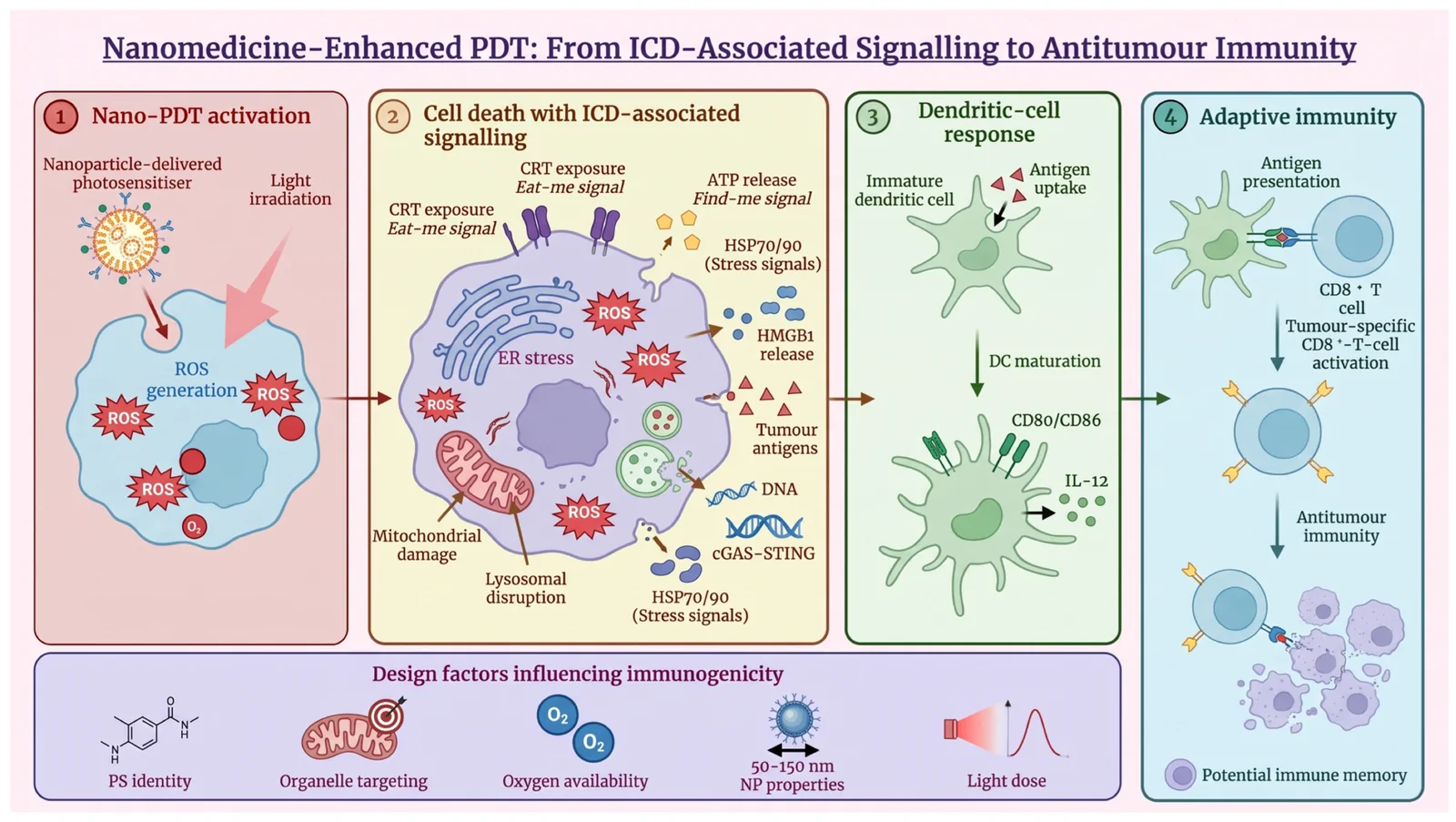
Nanomedicine-enhanced PDT promotes ICD-associated signalling and antitumour immune responses. Nano-PDT triggers danger-signal release, dendritic-cell activation and tumour-specific T-cell responses, influenced by nanoplatform design. Created in BioRender. Wilk, I. (2026) https://BioRender.com/cifhzxt, accessed on 25 September 2026.

Mature dendritic cells process tumour antigens and then present the antigen-derived peptides on major histocompatibility complex molecules. Local PDT damage is linked to tumour-specific T-cell priming by this. Oxidative stress was further linked to immunity by an Ir-MnII/III nanoparticle system, in hypoxic melanoma. This system generated photodynamic oxidative stress and was reported to induce both apoptosis and ferroptosis, with associated cGAS–STING activation [58]. Light-triggered ICD-associated signalling was similarly observed with Ce6-loaded γδ-T-cell exosome hybrids, which also promoted the maturation of dendritic cells and supported CD4+ and CD8+ T-cell responses directed against melanoma-associated antigens [43]. Thus, nanoparticle-enhanced PDT can link local ROS-mediated tumour damage with antigen presentation, as well as with the activation of tumour-specific T-cell responses, as shown in Figure 3.

### 5.3. How Nanoparticle Design Influences Immunogenicity

The immune effect of nanomedicine-enhanced PDT relies on more than the efficiency of tumour-cell killing. The relative contributions of Type I and Type II photochemical pathways are influenced by photosensitiser identity, the local substrate environment and oxygen availability, while the duration and magnitude of oxidative stress are also influenced by the light dose. Zinc-phthalocyanine PDT changed markers associated with ICD and immune-checkpoint expression in human melanoma cells. This shows that photodynamic conditions influence immune signalling as well as cytotoxicity [80]. As ROS act near their site of generation, subcellular localisation is equally important. A mitochondria-targeted pyropheophorbide-a nanophotosensitiser (Mito-PM) accumulated within the mitochondria of melanoma cells and generated ROS after irradiation. This caused mitochondrial dysfunction and ICD-associated danger signalling. This approach also stimulated antitumour immune activation in mice with melanoma, which shows that the immunogenic outcome of PDT can be influenced by subcellular photosensitiser localisation [81].

Cellular uptake, intracellular trafficking and the availability of the photosensitiser for activation are additionally influenced by nanoparticle size, surface chemistry and release kinetics [82]. Whether or not oxidative damage reaches immunogenic stress pathways, which include endoplasmic-reticulum stress, is influenced by these properties. ROS generation is also affected by oxygen availability, as tumour hypoxia can limit PDT reactions and reduce the induction of ICD-associated signals. In multifunctional systems the order of treatment also matters. Chemotherapy, PDT and immune modulation may reinforce or even interfere with each other, depending on when each element acts. In an aggressive melanoma model, a self-delivery chemo-PDT nanoplatform increased DAMP exposure and was also associated with dendritic-cell maturation and antitumour immune responses [83]. Immunity can therefore be improved by the addition of immunostimulatory agents. However, the success of the formulation should be judged using DAMP release, dendritic-cell activation and T-cell responses, rather than only tumour-cell viability.

### 5.4. Evidence in Skin Cancer Models

Evidence for ICD-associated signalling and functionally demonstrated antitumour immunity varies in strength across different types of skin cancer and experimental levels. For the purposes of this review, an antitumour effect beyond the treated lesion refers to activity at an untreated contralateral, distant or metastatic site, whereas systemic antitumour immunity additionally requires evidence of adaptive immune activation. Functional immune memory is reserved for studies demonstrating protection on tumour rechallenge; expansion of memory-phenotype T cells alone is considered supportive rather than definitive evidence. Melanoma currently has the most developed evidence base. ICD-associated signalling has been observed in vitro with a reduction-responsive Ce6/BMS-1 nanovehicle in B16F10 melanoma cells [84]. The same system boosted antitumour immune activity in animal models. Both the irradiated primary tumour and untreated contralateral B16F10 tumour were inhibited, providing evidence of an antitumour effect beyond the treated lesion [84]. A nanophotosensitiser targeted at the mitochondria also induced tumour regression and immune activation in mice with melanoma. When this system was combined with immune-checkpoint blockade, further benefit was observed [81]. This evidence base was recently extended by the TTO-Hemin@HA-PEI nanoplatform, which amplified PDT-associated oxidative damage and induced ICD-associated signalling and immune infiltration in melanoma models [85]. Programmed cell death protein 1 (PD-1)-cell-membrane-coated Ru (II)-Pt (IV) nanoparticles provide additional animal evidence. This multimodal system combined PDT, chemotherapy and checkpoint blockade and increased T-cell infiltration while suppressing proximal and distal or metastatic disease in mice, supporting systemic immune activation while preventing attribution of the effect to PDT alone [86]. Evidence in cSCC is less developed immunologically. Cu-Cy Nps@ZIF-8@HA nanoparticles produced apoptosis in A431 cells and suppressed A431 xenograft growth [48], while ALA/catalase-loaded PLGA nanoparticles delivered through soluble microneedles enhanced PDT in A431 cells and tumour-bearing mice [56]. More recently, PDA-Ce6-R837 nanoparticles induced ICD-associated signalling and tumour regression in a cSCC mouse model [87], although the platform combined PDT with photothermal therapy and Toll-like receptor 7 (TLR7) stimulation. In BCC, carbon-dot PDT after curettage has reached clinical evaluation [46], but immune endpoints were not assessed. Thus, the strongest evidence for immune activation extending beyond the treated tumour remains melanoma-derived, although disease-specific evidence is emerging in cSCC.

## 6. Immune Modulation and Emerging Photoimmunotherapy Strategies

The immune-modulation and photoimmunotherapy evidence discussed in this section is predominantly derived from melanoma models and should not be generalised to other cutaneous lesions without direct supporting evidence.

### 6.1. Effects on the Skin-Tumour Immune Microenvironment

In preclinical melanoma models, nanomedicine-enhanced PDT has been shown to remodel the tumour immune microenvironment and promote antitumour immune activation, sometimes alongside ICD-associated signalling. This occurs when the infiltration of immune cells is increased, while suppressive populations are reduced. Dendritic-cell maturation and intratumoral CD8+ T cells were increased in B16F10 melanoma by a tumour-responsive ARV/Ce6@RDP micellar platform. M2-like macrophages, regulatory T cells (Tregs) and myeloid-derived suppressor cells were also decreased by this approach [88]. Phagocytosis of treated tumour cells by dendritic cells and macrophages was enhanced by the same system, consistent with improved antigen-presentation potential, while increased IFN-γ-producing CD8+ and CD4+ T-cell populations indicated enhanced T-cell activation [88]. Cytokines may also promote inflammation. LaB_6_–polyethylene glycol (PEG)–folate nanoparticle PDT combined with anti-OX40 increased tumour necrosis factor-α (TNF-α), IFN-γ, IL-2 and IL-12, and promoted dendritic-cell maturation and CD4+ and CD8+ T-cell infiltration. M1-like macrophages were increased relative to M2-like macrophages, while regulatory T cells were reduced [89]. Serum levels of the immunosuppressive cytokines IL-10 and transforming growth factor-β (TGF-β) were also decreased, while natural killer (NK)-cell infiltration was increased [89]. In recent skin cancer nano-PDT studies neutrophils are likewise rarely quantified, so their contribution is insufficiently defined. Immune modulation may extend past the treated tumour. PD-1-membrane-coated Ru (II)-Pt (IV) nanoparticles enhanced the infiltration of CD4+ and CD8+ T-cells in mice with melanoma. Memory-phenotype CD4+ and CD8+ T-cell populations were also increased [86]. These findings support systemic immune activation and memory-associated T-cell phenotypes; however, without tumour-rechallenge evidence, they should not be interpreted as definitive evidence of durable protective immune memory.

### 6.2. Combination with Immune-Checkpoint Inhibitors

In melanoma models, combining nano-PDT with checkpoint inhibition has been investigated as a strategy by which to sustain PDT-induced antitumour immune responses. The tumour antigens and immunogenic signals produced by PDT help with the priming of dendritic cells, while the checkpoint blockade maintains the activity of T-cells that have just been activated. A combination of phototherapy and anti-programmed cell death protein 1 (PD-1) treatment in melanoma was brought together by TPC@ ovalbumin (OVA) porphyrin nanoparticles. The activation of T-cells and maturation of dendritic cells were increased by the nanoparticles, and the PD-1 blockade reduced immune suppression caused by the tumour [90]. Another strategy coated Ru (II)-Pt (IV) nanoparticles with engineered cell membranes expressing PD-1. The results showed that PDT increased T-cell infiltration in metastatic melanoma along with a sustained interception of programmed death-ligand 1 (PD-L1) [86]. The inhibition of PD-L1 is also possible through nanoparticle cargo. Reduction-responsive hyaluronic-acid nanoparticles which can co-deliver Ce6 and BMS-1 were able to release both agents in melanoma cells rich in glutathione. This spatially coupled PDT and PD-1/PD-L1 blockade [84]. The antibody was used by the anti-PD-L1 aggregation-induced emission (AIE) nanoparticles to target melanoma cells and also block PD-L1. Consequently, the accumulation of photosensitisers were increased and the growth of B16F10 melanoma was reduced [91]. Direct melanoma evidence for cytotoxic T-lymphocyte-associated protein 4 (CTLA-4) combinations remains limited. In B16F10 melanoma, redaporfin-PDT altered CD80, PD-L1 and CTLA-4 expression, although the addition of anti-CTLA-4 did not significantly improve tumour control over PDT alone under the conditions tested [92]. The same study also evaluated noncutaneous CT26 colon carcinoma and 4T1 mammary carcinoma models; findings from these models are considered here as comparative evidence and are not extrapolated to cutaneous disease. CTLA-4 is supported as an important therapeutic target in melanoma by clinical evidence [93]. Lymphocyte-activation gene 3 (LAG-3) is also clinically validated in melanoma [93]; however, convincing combinations targeting LAG-3, T-cell immunoglobulin and mucin-domain containing-3 (TIM-3) or T-cell immunoreceptor with immunoglobulin and immunoreceptor tyrosine-based inhibitory motif (ITIM) domains (TIGIT) have not yet been reported. Newer nanoplatforms are thus being designed in such a way that they combine checkpoint inhibition with PDT-induced immune activation.

### 6.3. Nanoparticle Delivery of Immune Adjuvants

Nanoparticle delivery can improve photoimmunotherapy by concentrating immune adjuvants within melanoma, all while keeping systemic exposure to a minimum. It can be particularly useful after PDT when tumour antigens are released, creating an opportunity for local innate stimulation to enhance the activation of dendritic cells. A soluble microneedle vaccine co-delivered liposomal Trp2 antigen, the Toll-like receptor 7/8 agonist R848 and IL-12 messenger RNA (mRNA), which linked antigen presentation with cytokine-stimulated T-cell activation [94]. Transdermal LA-Mn-cGAMP nanoassemblies served as a direct route for the STING agonist cGAMP, increasing STING activation and reducing melanoma-associated immunosuppression [95]. The local production of cytokines from delivered nucleic acids is also possible. In B16F10 melanoma, poly (β-amino ester) nanoparticles co-delivering plasmids encoding costimulatory molecules and immunostimulatory cytokines increased immune-cell infiltration and shifted adaptive and innate immune populations towards immunostimulatory phenotypes. Combinations including 4-1BB ligand (4-1BBL)/IL-12 and CD80/IL-12 showed prominent activity [96]. Melanoma-selective PEG-PLGA/1,2-dioleoyl-3-trimethylammonium-propane (DOTAP) nanoparticles co-encoding the C-X-C motif chemokine ligand 9 (CXCL9) and an anti-PD-L1 single-chain variable fragment induced tumour-cell-specific expression, generating a CXCL9 gradient that recruited T cells while simultaneously promoting their activation through local PD-L1 blockade [97]. Suppressive metabolic pathways are another area of concern. A study that used a purpurin-18 liposomal photoimmunotherapy system to deliver epacadostat, an inhibitor of indoleamine 2,3-dioxygenase, recorded better dendritic cell maturation and cytotoxic T-cell activation while observing a reduction in regulatory T cells [98]. Myeloid targeting is also emerging. gp100/R848 nanovesicles were able to shift tumour-associated macrophages from the M2 state to the M1 state and also reduce myeloid-derived suppressor cells (MDSCs) [99]. These nanoplatforms together develop the capabilities of nano-PDT beyond tumour killing. They guide it towards spatially controlled immune activation and the recruitment and relief of local immunosuppression while possibly lowering the toxicity of systemic adjuvants.

### 6.4. Nano-PDT as an In Situ Cancer Vaccination Strategy

Preclinical studies, predominantly in melanoma, have investigated nano-PDT as an in-situ vaccination strategy by coupling photodynamic tumour destruction with antigen release and immunostimulatory signalling. In a noncutaneous MB49 bladder-cancer model, an antigen-capturing nanoplatform was designed to bind tumour antigens released after PDT and deliver them to dendritic cells, increasing dendritic-cell maturation and T-cell activation [100]. This study is considered here as mechanistic evidence for antigen capture rather than direct evidence in cutaneous disease. Similarly, tumour antigens released following combined PDT/PTT in a postoperative B16F10 melanoma model were captured by a photoresponsive hydrogel, which acted as an antigen reservoir and recruited dendritic cells to promote antitumour immunity [101]. Thus, tumour material that is usually short-lived can be preserved by these approaches and could improve cross-presentation rather than relying on antigen release alone. Major histocompatibility complex class I (MHC-I)-enriched melanoma membrane-coated Cu@ferrihydrite nanoparticles also promoted dendritic-cell maturation and activated CD8+ and CD4+ T cells. Systemic activation of these immune populations in lymphoid organs and the lungs was associated with prophylactic protection against pulmonary metastasis [102]. A light-triggered Fe-TCPP-R848-PEG nanozyme combining PDT with ferroptosis-promoting activity and immune stimulation inhibited distant B16F10 tumours and provided protection against subsequent tumour rechallenge, supplying functional evidence of immune memory [103]. Activity similar to vaccination is not an automatic consequence of PDT. A NanoCRISPR/haem oxygenase-1 (HO-1) scaffold reduced tumour tolerance to PDT-associated oxidative stress and enhanced antigen generation and T-cell activation. When combined with anti-PD-L1 therapy, the platform produced durable protection against melanoma rechallenge and reduced metastatic progression, supporting functional immune memory [104]. Therefore, prolonged antitumour protection requires more than tumour-cell death or ICD-associated signalling alone; effective antigen presentation and functional adaptive immunity are also required.

### 6.5. Multimodal Nanomedicine-Based Combination Therapy

**Figure 4:**
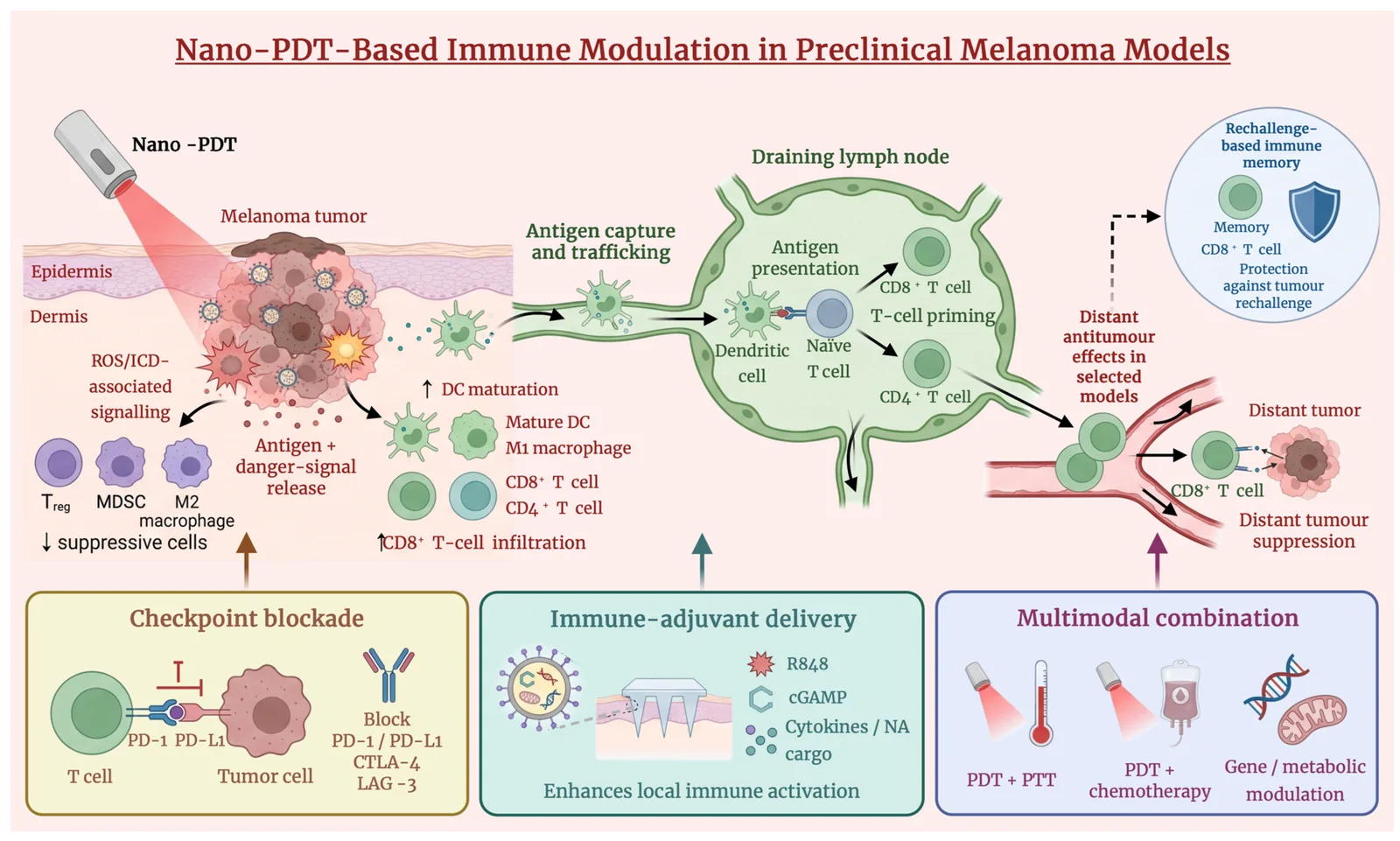
Nano-PDT-mediated immune modulation demonstrated predominantly in preclinical melanoma models. Representative nano-PDT-based strategies have promoted local tumour control and T-cell activation. Antitumour effects at untreated distant tumours have been demonstrated in selected models, while protective immune memory is supported where tumour-rechallenge experiments were performed. Created in BioRender. Wilk, I. (2026) https://BioRender.com/mt3mo70, accessed on 25 September 2026.

PDT can be combined with mechanistically distinct therapeutic components in multimodal nanomedicine. Where PTT, CDT, chemotherapy or ferroptosis-promoting mechanisms contribute to treatment, the overall antitumour response cannot be attributed to PDT alone. A system combined magnetic microbubbles with ICG-loaded liposomes in melanoma. This integrated PDT with mild photothermal therapy. In melanoma models, tumour suppression was improved by this system as the resulting heat and ROS depleted glutathione and promoted ferroptosis [105]. The effects of chemotherapy, PDT, photothermal and chemodynamic therapy were integrated in B16F10 cells and melanoma-bearing mice by a separate MIL-101-NH_2_(Fe) platform. Doxorubicin and indocyanine green were also co-delivered in this approach [106]. Another approach is offered by gene editing. Tumour tolerance to PDT-associated oxidative stress was reduced by a NanoCRISPR/HO-1 scaffold. Combined treatment with anti-PD-L1 further promoted antitumour immunity and protection against tumour rechallenge, supporting durable immune memory in this melanoma model [104]. Oxidative treatment can also be accompanied by anti-angiogenic activity. Ce6-modified Fe-doped carbon dots supported ferroptosis while suppressing angiogenesis and migration in B16 melanoma. PDT was linked with vascular disruption by this system [107]. Another approach is offered by metabolic modulation. Transdermal 2-deoxy-D-glucose (2-DG) DES-MON@Ce6 inhibited glycolysis, reduced hypoxia and enhanced subsequent Ce6-mediated PDT in melanoma [49]. These examples illustrate how PDT can function as one component of multimodal treatment, while the contributions of PTT, CDT, chemotherapy, metabolic modulation and ferroptosis-promoting mechanisms should be distinguished when interpreting therapeutic efficacy. There is little evidence for recent nano-PDT combinations with radiotherapy in skin cancer and most of these are still preclinical. Table 3 and Figure 4 summarise the representative nanomedicine-enhanced PDT strategies for immune modulation and multimodal therapy.

## 7. Disease- and Lesion-Specific Clinical and Preclinical Evidence for Nanomedicine-Enhanced PDT

To clarify the translational status of these approaches, the evidence is considered separately for AK, BCC, cSCC/Bowen disease and melanoma, with clinically established conventional PDT distinguished from clinically evaluated nanomedicine-assisted PDT and emerging preclinical nano-PDT, ICD and photoimmunotherapy strategies.

### 7.1. Actinic Keratosis

Clinically established PDT. Actinic keratosis (AK) is a UV-associated premalignant keratinocytic lesion and potential precursor of cutaneous squamous-cell carcinoma (cSCC). For patients with multiple lesions or field cancerization, current European guidance includes PDT among the recommended field-directed treatment approaches [108]. Clinical nanomedicine-assisted PDT. A clinically established example of nanoformulation-assisted PDT in AK is BF-200 ALA, a nanoemulsion-based formulation of 5-aminolevulinic acid developed to improve ALA stability and cutaneous penetration [109]. In a multicentre phase III trial involving 600 patients, BF-200 ALA-PDT achieved complete patient clearance in 78.2% of patients, compared with 64.2% for methyl aminolevulinate-PDT and 17.1% for placebo [109]. In a subsequent phase III field-directed study, complete patient and lesion clearance rates after a maximum of two treatments were 91% and 94.3%, respectively, compared with 22% and 32.9% with placebo [110]. Longer-term follow-up also showed sustained clinical benefit at 12 months [111]. Immune and photoimmunotherapy evidence. These studies therefore provide direct human evidence for nanoformulation-assisted ALA-PDT in AK; however, their evidence concerns clinical efficacy, safety and cosmetic outcomes rather than ICD, systemic antitumour immunity or photoimmunotherapy. Consequently, the immune effects demonstrated with more complex nanoplatforms in melanoma should not be extrapolated to AK without direct supporting evidence.

### 7.2. Basal Cell Carcinoma

Clinically established PDT. Photodynamic therapy is an established non-surgical option for selected superficial and other low-risk basal cell carcinomas (BCCs). 

Clinical nanomedicine-assisted PDT. Nanotechnology has entered this setting clinically through BF-200 ALA, a nanoemulsion formulation of 5-aminolevulinic acid. In a randomised phase III trial involving 281 patients with nonaggressive BCC, BF-200 ALA-PDT achieved complete patient responses in 93.4% of patients compared with 91.8% with MAL-PDT, establishing non-inferiority [112]. Long-term data are more cautious: in a separate randomised study of low-risk BCC, the five-year cumulative response rate was 54.6% with BF-200 ALA, compared with 71.9% with MAL [113]. More recently, carbon-dot PDT following curettage was evaluated clinically in 22 patients with 27 BCC or Bowen-disease lesions, with complete responses reported for several BCC subtypes and no irradiation-associated pain [46]. However, this study lacked a conventional PDT comparator and all lesions underwent curettage, limiting the attribution of efficacy to the nanoplatform alone. 

Immune and photoimmunotherapy evidence. Overall, BCC has direct human evidence for nanotechnology-assisted PDT, but current studies primarily address local tumour response and tolerability rather than ICD or systemic antitumour immunity; melanoma-derived immune effects therefore cannot yet be generalised to BCC.

### 7.3. Cutaneous Squamous Cell Carcinoma and Bowen Disease

Clinically established PDT. Bowen disease represents cutaneous squamous cell carcinoma (cSCC) in situ and is an established indication for topical PDT, although long-term disease control may be less favourable than with surgical excision. In a recent multicentre randomised trial, the four-year cumulative probability of tumour-free survival was 82.7% with MAL-PDT compared with 97.5% after surgical excision [114].

Clinical nanomedicine-assisted PDT. Nanomedicine-specific clinical evidence is more limited; carbon-dot PDT following curettage produced only partial responses in Bowen-disease lesions in the recent clinical study described above [46].

Emerging preclinical nano-PDT and immune strategies. In invasive cSCC models, ALA- and catalase-loaded PLGA nanoparticles incorporated into soluble microneedles improved transdermal delivery, oxygen availability and PDT efficacy in A431 cells and tumour-bearing mice [56]. More recently, a polydopamine nanoplatform co-delivering Ce6 and the TLR7 agonist R837 induced ICD-associated signalling and complete tumour regression in a cSCC mouse model [87]. However, this system combined PDT with photothermal therapy and immune stimulation, so its antitumour effect cannot be attributed to PDT alone. Thus, cSCC now has emerging direct evidence for both nanomedicine-enhanced PDT and immune modulation, but clinical validation beyond Bowen disease remains limited.

### 7.4. Melanoma

Clinical PDT status. Unlike AK, superficial BCC and Bowen disease, PDT is not an established routine treatment for melanoma; current clinical melanoma treatment guidelines instead focus on surgical excision, immune-checkpoint inhibition and, for selected BRAF-mutant disease, BRAF/MEK-targeted therapy [115].

Emerging preclinical nano-PDT and photoimmunotherapy. Melanoma provides the most developed mechanistic evidence for nanomedicine-enhanced PDT among the malignant cutaneous models reviewed here, although this evidence remains predominantly preclinical. In A375 and SK-Mel-28 cells, PDA-Ce6-R837 nanoparticles induced calreticulin exposure and extracellular ATP and HMGB1 release, together with enhanced dendritic-cell maturation [77]. In B16F10 melanoma models, reduction-responsive Ce6/BMS-1 nanoparticles combined PDT-induced ICD-associated signalling with PD-1/PD-L1 inhibition and produced antitumour effects in both treated and untreated contralateral tumours [84]. More recently, PD-1-cell-membrane-coated Ru(II)-Pt(IV) nanoparticles combined PDT, chemotherapy and checkpoint inhibition to increase T-cell infiltration and suppress primary and metastatic melanoma in mice [86]. Because this was a multimodal platform, its effects cannot be attributed to PDT alone. Thus, melanoma represents the most mechanistically developed but predominantly preclinical setting for nano-PDT, and these findings should not be extrapolated to AK, BCC or cSCC without direct supporting evidence. The disease-specific distribution of in vitro, animal and human evidence for nanomedicine-enhanced PDT is summarised in Table 4.

## 8. Translational Challenges and Clinical Prospects

### 8.1. Current Level of Evidence

As summarised in Table 4, the maturity of evidence differs substantially between cutaneous disease types. Conventional topical PDT is clinically established for AK, selected superficial BCC and Bowen disease, whereas the translational status of nanomedicine-enhanced PDT is more heterogeneous. Human evidence in AK and BCC primarily concerns nanoformulation-assisted local PDT, while advanced multifunctional nano-PDT, ICD-associated and photoimmunotherapy strategies remain predominantly preclinical and melanoma-based. Limited clinical investigation has also been reported for carbon-dot PDT in patients with BCC or Bowen disease [46]. Thus, increasing experimental sophistication should not be interpreted as equivalent clinical maturity. Translation of more complex nanoplatforms remains limited by manufacturing reproducibility, safety and regulatory requirements [73].

### 8.2. Localised Versus Systemic Nano-PDT and Dermatological Treatment Barriers

The direct accessibility of many cutaneous lesions creates an important distinction between localised and systemic nano-PDT. Topical, intradermal and microneedle-based systems can deliver photosensitisers directly to the treatment site while limiting systemic exposure, making them particularly relevant to superficial lesions. Nanoformulations may improve photosensitiser stability, cutaneous retention and penetration, while microneedles can partly bypass the stratum corneum [33,55,56]. By contrast, systemically administered nanoparticles remain dependent on circulation, tumour accumulation and tissue distribution, and therefore introduce additional concerns regarding off-target biodistribution and clearance. In the skin-cancer literature reviewed here, these more complex systemic approaches remain predominantly preclinical and are especially represented in melanoma models.

The clinical benefit of nanotechnology is nevertheless constrained by lesion-specific factors. Increased lesion thickness, tumour depth and keratinisation can restrict topical photosensitiser delivery, while limited penetration of activating light remains an important barrier that improved drug delivery alone cannot overcome [116]. Pigmentation represents an additional limitation in melanoma because it can reduce effective light delivery, although optical-clearing approaches have shown preclinical potential [24]. Treatment-associated pain may also vary according to formulation and irradiation conditions; notably, no irradiation-associated pain was reported in the clinical carbon-dot study [46]. Improved local delivery and intratumoral distribution may reduce incomplete treatment, but whether nano-PDT reduces long-term recurrence compared with established therapies remains unresolved. Thus, nanotechnology can realistically improve photosensitiser formulation, skin penetration, local retention and, in selected systems, oxygen availability, whereas tumour depth, light penetration, pigmentation and durable recurrence control remain important clinical barriers.

### 8.3. Nanomaterial Safety and Pharmacology

The safety of nanomaterials needs to be assessed beyond short-term tumour response. The composition, size, surface chemistry, dose and persistence of nanoparticles significantly influence toxicity, while poorly degradable materials may persist in tissues or healthy organs [72]. These pharmacological considerations are route dependent. Systemic and poorly degradable nanoplatforms require particular attention to organ biodistribution, metabolism and clearance. In contrast, locally administered cutaneous systems require assessment of skin retention, local toxicity and potential systemic distribution [71]. After repeated exposure in particular, the complement system, inflammatory pathways or adaptive immune responses may be activated by carrier materials [117]. Thus, careful assessment of biodegradation products will be needed, as well as long-term toxicity and pharmacokinetics. The clearance, organ accumulation and immune recognition of the nanocarrier may change during subsequent administrations. Repeated-dose studies are therefore particularly important [117].

### 8.4. Manufacturing, Standardisation and Regulation

The production of nanoparticles must be reproducible and scalable to support clinical translation. Between different batches, particle size, surface properties and photosensitiser loading must remain consistent. Sterility, stability and storage also require careful control [71]. Manufacturing under good manufacturing practice should include defined critical quality attributes, in-process testing and appropriate analytical methods [71]. Laboratory formulations can be difficult to reproduce because scale-up may alter mixing, loading efficiency, aggregation and release. Multifunctional systems introduce additional challenges, as each targeting ligand, imaging agent or immune component adds manufacturing and quality-control requirements, while products combining drug, nanomaterial or diagnostic functions may also face greater regulatory complexity [74].

These translational challenges vary considerably between nanoplatforms. Relatively simple organic carriers and skin-directed systems, including topical formulations and microneedles, may offer translational advantages by reducing systemic exposure and limiting concerns related to off-target biodistribution. In contrast, poorly degradable inorganic nanoparticles require more extensive evaluation of tissue persistence, organ accumulation and clearance [71,72,117]. Highly multifunctional targeted, oxygen-generating and theranostic systems may provide substantial experimental benefits, but their greater structural and functional complexity can further increase manufacturing and regulatory demands [71,74]. Accordingly, experimentally sophisticated platforms should not automatically be regarded as the most clinically translatable, and clinical potential will depend on achieving an appropriate balance between therapeutic functionality, safety, manufacturability and regulatory feasibility.

### 8.5. Clinical Study Design and Patient Selection

Drug and light parameters should also be made consistent in clinical studies. Photosensitiser dose, nanoparticle dose, drug-to-light interval, wavelength and fluence should be reported in protocols. Fluence rate and follow-up duration should also be reported. As it is possible for tissue absorption and scattering to vary patient to patient, calibrated fluence-rate measurement is highlighted in modern PDT dosimetry guidance [118]. For oxygen-dependent PDT, oxygen monitoring should be added where possible. Rather than just the size of the tumour alone, defined endpoints should be measured in immune-focused trials. Potential biomarkers for patient selection are tumour hypoxia, PD-L1 levels and the degree of immune-cell infiltration. All of these have been observed to be altered by recent melanoma nanoplatforms [55,58]. Patients who are most likely to benefit from treatment with nanomedicine-enhanced PDT may be identified by photosensitiser accumulation, tumour thickness and vascularity.

## 9. Discussion and Future Perspectives

### 9.1. Discussion

A major translational imbalance identified in this review becomes apparent when the evidence is considered by disease type. In AK, selected superficial BCC and Bowen disease, conventional PDT is already clinically established [8,17,18,19,20], while human nanomedicine evidence primarily concerns nanoformulation-assisted local PDT, including BF-200 ALA in AK and BCC and limited carbon-dot PDT investigation in BCC and Bowen disease [46,109,112,113]. By contrast, evidence for nanomedicine-enhanced PDT in invasive cSCC remains predominantly preclinical, despite emerging experimental data on improved delivery, tumour control and immune modulation [48,56,87]. Thus, the presence of clinical PDT evidence in superficial or in situ disease should not be interpreted as equivalent clinical maturity for advanced nano-PDT or photoimmunotherapy approaches.

Melanoma shows a different pattern. Although PDT is not an established routine melanoma treatment [115], it provides the most extensive mechanistic and immune-focused evidence for advanced nano-PDT among the cutaneous diseases reviewed here, including ICD-associated signalling [77], checkpoint-based combinations [84,86] and antitumour effects at untreated distant or metastatic sites in selected preclinical models [84,86]. Functional immune memory has been demonstrated in a smaller subset of multimodal melanoma studies that included tumour-rechallenge experiments [103,104]. However, these findings remain predominantly preclinical and should not be extrapolated to AK, BCC or cSCC without direct supporting evidence. Overall, the translational maturity of nanomedicine-enhanced PDT is therefore disease-dependent: clinically established PDT and human nanoformulation evidence are concentrated mainly in superficial cutaneous lesions, whereas the most mechanistically developed immune-modulating and photoimmunotherapy strategies remain largely melanoma-derived and experimental.

### 9.2. Future Perspectives

In the future, platforms for nanomedicine-enhanced PDT in cutaneous oncology should prioritise systems that have a real potential for clinical use, instead of creating more unnecessarily complicated platform designs. Biodegradable nanocarriers with reproducible composition, scalable manufacture and clear pharmacological behaviour are much more likely to be valuable in the future than multifunctional nanoplatforms which are overly complex. Only when each element provides a clear therapeutic benefit should multifunctionality be retained. Components that improve tumour selectivity, efficiency of photochemistry or immune activation are especially valuable in this regard [71,74].

Maintaining activity under hypoxic conditions should be a priority for future photosensitisers, as this currently limits PDT. Approaches that favour Type I photochemistry or reduce dependence on oxygen would be especially valuable [55,67]. Designs should also more intentionally use organelle targeting to control the quality of oxidative stress and increase the probability that tumour-cell death is accompanied by ICD-associated signalling and functional immune activation, rather than simply amplifying tumour-cell damage [38,81]. Tumour characteristics such as hypoxia, photosensitiser uptake and PD-L1 expression could also be increasingly used to guide treatment selection, as the field develops [55,58]. Photosensitisers and immune combinations may be matched to individual tumours more rationally in this way, and patients who are most likely to benefit from photoimmunotherapy may be identified.

Many cutaneous lesions and tumours are easily accessible, providing a practical opportunity to translate these advances. Nanomedicine-enhanced PDT approaches should make use of this in the future by supporting local approaches. These should include topical and microneedle delivery that can keep systemic treatment limited whilst improving tumour exposure [46,55,56]. Treatment should also become more closely combined with imaging and dosimetry. This would allow for real-time assessment of photosensitiser accumulation, oxygenation and light delivery [47,69]. The most promising platforms will ultimately be the ones that combine biological effectiveness with simplicity, reproducibility and a clinical benefit that is assessable. Standardised treatment protocols, robust safety assessment and well-designed clinical trials that include immune outcomes, recurrence and longer-term treatment control should be used for their evaluation in the future [71,118].

## 10. Conclusions

By managing several limitations and offering a chance for therapy that is more precise and immunologically active, nanomedicine is reshaping conventional PDT treatment. Nanocarriers can improve the delivery, tumour selectivity and photochemical performance of photosensitisers, and this has been shown in recent advances. The availability of oxygen, intracellular localisation and pattern of tumour-cell death may also be influenced by nanocarriers. Determining when local photodynamic injury progresses from ICD-associated signalling to functional antitumour immunity is of particular importance. This creates a foundation for combining nanomedicine-enhanced PDT with immune-checkpoint blockade and other immunomodulatory strategies. Selected preclinical melanoma studies indicate that nano-PDT-based combinations can extend antitumour effects beyond the treated lesion, while durable immune memory has been functionally demonstrated only in studies using tumour rechallenge. Whether these effects translate into long-term systemic tumour control clinically remains unresolved.

Nanomedicine-enhanced PDT is still very much in experimental development rather than being routinely clinically applied. The most developed immune and photoimmunotherapy evidence remains predominantly melanoma-derived and preclinical. Human nanomedicine evidence in AK and BCC mainly concerns local nanoformulation-assisted PDT. Disease-specific clinical validation is therefore required before these immune effects can be generalised across cutaneous oncology. Increasing platform complexity may create new challenges instead of solving current ones. Therefore, the field seems to be moving from proof-of-concept nanoplatform development towards assessing if clinical benefit can come from these experimental advantages. The key question is whether these nanomedicine-associated photodynamic and immune advantages can translate into reliable tumour control and durable clinical benefit within each specific cutaneous disease context.

## Figures and Tables

**Table 1:** Representative experimental evidence for organic nanocarrier-enhanced PDT in skin cancer, predominantly from melanoma models. Studies are presented in the order in which they are discussed in Section 4.1 to facilitate cross-referencing between the narrative and the table.

Ref.	Carrier Class	Model/Formulation	Comparator/PDT Conditions	Principal Outcome	Evidence Level	Comparative Advantage/Limitation
[36]	Polymeric–lipid hybrid liposome	B16-F10 melanoma; CUR/AgNP-loaded DPPC–F127 liposomes	CUR formulations ± AgNPs/PDT; intratumoral in vivo	Increased skin CUR retention; reduced tumour growth and prolonged survival	Ex vivo + in vitro + in vivo	Enhanced skin retention and tumour control; intratumoral delivery and AgNP activity limit attribution to carrier-mediated PDT
[37]	Polymeric NPs + microneedles	A375 melanoma and A431 cSCC; ALA–tea-polyphenol NPs	ALA@MNs/TP-NP@MNs; 635 nm, 80 mW/cm^2^, 5 min	Viability 15.3% (A375) and 30.5% (A431); enhanced tumour suppression	In vitro + in vivo	Improved transdermal delivery at reduced ALA dose; active tea polyphenols confound PDT-specific effects
[38]	ER-targeted peptide micelle	A375 melanoma; P-1-ERT@Rap	Relevant drug/light controls; 650 nm, 1 W/cm^2^, 5 min in vivo	Increased tumour accumulation and inhibited xenograft growth	In vitro + in vivo mechanistic/tumour control	pH-responsive ER-targeted delivery; rapamycin activity complicates attribution to PDT alone
[39]	Nanoemulsion	B16-F10 and HaCaT; chlorophyll nanoemulsion	Free chlorophyll/blank/dark controls; 660 nm, 108 J/cm^2^, 15 min	IC_50_ 313.4 vs. 545.2 µM in B16-F10 vs. HaCaT	In vitro selectivity	Normal-cell comparison and 365-day stability; no in vivo validation
[40]	Nanostructured lipid carrier	A2058 melanoma; NLC-PpIX	Dark vs. light; 420 nm	~96% inhibition at 200 µg/mL; no dark toxicity	In vitro phototoxicity	Improved aqueous delivery with strong phototoxicity; no normal-cell or in vivo validation
[41]	Protein-based hybrid	A375 melanoma; Pc–BSA–Au	Free Pc/Pc-BSA; 680 nm, 10 J/cm^2^, 15 min	Survival 23.1% vs. 48.7% with free Pc	Comparative in vitro phototoxicity	Enhanced phototoxicity versus free Pc; no normal-cell or in vivo validation
[42]	Solid lipid NP	B16-F10 + dendritic cells; AlPc-SLNs	Blank SLNs/MTX; 660 nm, 25.9 J/cm^2^, 10 min	ROS/DAMP changes and dendritic-cell activation	In vitro DAMP/DC-activation evidence	Demonstrates ICD-associated DAMP changes and DC activation; no functional in vivo antitumour immunity demonstrated
[43]	Liposome–exosome hybrid	A375 melanoma; Ce6 liposome–γδ-T-exosome	Ce6 liposomes/exosomes; 650 nm	Tumour accumulation 2.29× free Ce6; tumour suppression and immune activation	In vivo tumour + immune evidence	Advanced in vivo immune evidence; exosome activity confounds PDT-specific effects
[44]	Dendrimer	Healthy BALB/c mice; Rose-Bengal dendrimer	PBS/free RB/unloaded carrier; no PDT	No significant systemic toxicity over 4 weeks	In vivo safety only	Supports short-term tolerability; no tumour model or PDT efficacy

**Table 2:** Representative recent nanomedicine strategies for enhanced PDT in skin cancer, predominantly from melanoma models.

Design Principle	Representative Nanoplatform	Primary Contribution to PDT	Disease/Model-Specific Evidence	Key Translational Consideration
Organic nanocarrier [36]	Curcumin/silver-loaded polymeric–lipid hybrid liposomes	Improves photosensitiser retention and delivery	Enhanced skin retention and suppressed B16-F10 melanoma growth	Reproducible particle characteristics, photosensitiser loading, stability and scale-up require standardisation [71]
Inorganic nanoplatform [45]	PpIX-loaded mesoporous silica-coated spicules	Improves penetration into deeper skin layers	Eradicated primary melanoma in mice without recurrence during 60-day follow-up	Long-term nanomaterial safety, biodistribution and persistence require evaluation [72]
Active tumour targeting [53]	Anti-MIA-functionalised Au–ZnPcS4 nanocarrier	Antibody-mediated melanoma targeting	Increased photosensitiser bioavailability, ROS generation and phototoxicity in melanoma models	Gold-based systems require consideration of long-term accumulation, while antibody functionalisation increases manufacturing complexity [73,74]
Skin-directed delivery [55]	ZnPc-containing DNA-nanoflower microneedle patch	Bypasses the skin barrier and improves local delivery	Relieved hypoxia, suppressed B16F10 melanoma and enhanced antitumour immunity	Local microneedle delivery avoids reliance on systemic administration, but reproducible manufacture requires standardisation [55,71]
Stimuli-responsive delivery [58]	pH–light-responsive Ir–MnII/III nanoparticles	Acid-triggered activation followed by light-induced ROS generation	Generated oxygen and light-induced ROS; apoptosis and ferroptosis were reported as cell-death outcomes in orthotopic melanoma	Multicomponent responsive designs increase manufacturing and regulatory complexity and require long-term safety assessment [72,74]
Organelle targeting [38]	ER-targeted self-assembling peptide nanoplatform	Localises photodynamic damage to the endoplasmic reticulum	Increased tumour accumulation and PDT efficacy in A375 melanoma xenografts	Targeted multifunctional design requires reproducible manufacture and physicochemical characterisation [74]
Oxygen modulation [62]	MnO_2_-containing magnetic nanocomposite	Catalytically generates O_2_ from endogenous H_2_O_2_	Increased ROS and suppressed B16-F10 melanoma growth and metastasis	Biodistribution, persistence and long-term nanomaterial safety require evaluation [72]
ROS amplification [67]	PFBT/PCBM heterojunction nanoparticles	Generates radical ROS through Type I photochemistry and singlet oxygen through Type II photochemistry; melanin accelerates photoredox cycling	Produced strong antitumour activity in melanoma cells and tumour-bearing mice	Long-term safety, biodistribution and standardised physicochemical characterisation remain necessary [71,72]
MRI-guided theranostics [69]	SPIO/ICG nanomicelles	Tracks tumour accumulation and guides irradiation timing	MRI identified peak accumulation before PTT/PDT in B16-F10 melanoma	Combining imaging and therapeutic functions increases manufacturing and regulatory complexity [74]
Treatment monitoring [47]	UCNP-assisted Rose-Bengal PDT	Enables oxygen/ROS monitoring during treatment	Real-time assessment of oxygen consumption and ROS dynamics in A375 melanoma	Integrated monitoring and therapeutic functions increase regulatory complexity, while long-term nanomaterial safety requires evaluation [72,74]

**Table 3:** Representative nanomedicine-enhanced PDT strategies for immune modulation and multimodal therapy in preclinical skin-cancer models, predominantly melanoma.

Strategy	Representative System	Main Mechanism	Key Outcome	Model
TME remodelling	ARV/Ce6@RDP micelles [88]	Increased DC/T-cell activity, decreased Tregs, MDSCs and M2 macrophages	More immunologically active tumour microenvironment	B16F10 melanoma
Checkpoint blockade	Ce6/BMS-1 nanoparticles [84]	PDT + PD-1/PD-L1 inhibition	ICD-associated signalling, T-cell infiltration and suppression of an untreated contralateral tumour	B16F10 melanoma, bilateral tumours
Immune-adjuvant delivery	Trp2/R848/IL-12 mRNA microneedles [94]	Antigen delivery with TLR7/8 and cytokine stimulation	Enhanced DC and T-cell activation	Melanoma
In situ vaccination	TCPP-R848 nanozyme [103]	PDT + ferroptosis-promoting activity + immune stimulation	Distant tumour suppression and protection on tumour rechallenge	B16F10 melanoma
Multimodal phototherapy	ICG-loaded liposomes/magnetic microbubbles [105]	PDT + mild PTT; glutathione (GSH) depletion with ferroptosis as a downstream cell-death mechanism	Improved tumour suppression	Melanoma models
Gene/metabolic modulation	NanoCRISPR/HO-1 [104], 2-DG DES-MON@Ce6 [49]	Reduced ROS tolerance or glycolysis/hypoxia	Enhanced PDT and prolonged antitumour effects	Melanoma models

**Table 4:** Disease-specific clinical status and level of evidence for nanomedicine-enhanced photodynamic therapy in cutaneous oncology.

Lesion/Tumour Type	Established Conventional PDT Status	In Vitro Nano-PDT Evidence	Animal Nano-PDT Evidence	Human Nanomedicine-Assisted PDT Evidence	Immune/ICD-Associated Evidence	Main Evidence Gap
Actinic keratosis (AK; premalignant lesion)	Established, including field-directed treatment [8,17]	Limited/not a major focus	Limited/not a major focus	Phase III evidence for BF-200 ALA nanoemulsion PDT, with follow-up studies [109,110,111]	Not assessed in the cited clinical studies	Human evidence primarily addresses local efficacy, safety and cosmetic outcomes rather than immune effects
Basal cell carcinoma (BCC)	Established option for selected superficial/low-risk BCC [8,18,19]	Limited	Limited	Randomised evidence for BF-200 ALA-PDT; early clinical carbon-dot PDT evidence [46,112,113]	Not assessed in the cited clinical studies	Advanced immune-modulating nano-PDT lacks clinical validation
cSCC/Bowen disease	Established for Bowen disease/SCC in situ [8,20]	Direct A431/cSCC evidence: multimodal PDT/CDT [48] and nano-PDT [56]	Tumour-control evidence from multimodal PDT/CDT [48] and nano-PDT [56]	Limited; carbon-dot PDT evaluated clinically in Bowen disease [46]	Preclinical ICD-associated signalling and immune-modulating evidence [87]	Evidence for invasive cSCC remains predominantly preclinical
Melanoma	Not an established routine PDT indication [115]	Extensive across A375, SK-Mel-28 and B16/B16F10 models [77,84,86]	Extensive tumour-control and immune-response evidence [77,84,86]	No direct clinical nanomedicine-enhanced PDT evidence identified in this review	Most developed preclinical ICD-associated/photoimmunotherapy evidence [77,84,86]	Extensive mechanistic evidence but limited clinical translation

## Data Availability

No new data were created or analyzed in this study. Data sharing is not applicable to this article.

## Supplementary Materials

The following supporting information can be downloaded at https://www.mdpi.com/article/10.3390/ijms27198723/s1.
