# Supplementary material for: Recent Advances in Nanomedicine-Enhanced Photodynamic Therapy in Cutaneous Oncology: Melanoma-Dominant Evidence for Immunogenic Cell Death and Photoimmunotherapy
Source: Int J Mol Sci. 2026 Sep 29;27(19):8723. doi: 10.3390/ijms27198723 (PMC13665337; doi:10.3390/ijms27198723)
Supplement: Supplementary file 1 [file ijms-27-08723-s001.zip › ijms-4568866-supplementary.pdf]

Supplementary Table S1. Database-specific search strategies used for literature identification

The expanded search strings are reproduced verbatim from the PubMed and PubMed Central (PMC) Search Details following automatic term mapping and were not manually modified. "AND" was used to combine major search concepts and "OR" to combine synonymous or related terms. Field tags included MeSH Terms and All Fields where applicable. No additional filters or limits were applied at the database-search stage.

Database	Search concept	Exact search string	Filters/limits
PubMed	nanomedicine photodynamic therapy skin cancer	("nanomedicinal"[All Fields] OR "nanomedicine"[MeSH Terms] OR "nanomedicine"[All Fields] OR "nanomedicines"[All Fields]) AND ("photochemotherapy"[MeSH Terms] OR "photochemotherapy"[All Fields] OR ("photodynamic"[All Fields] AND "therapy"[All Fields]) OR "photodynamic therapy"[All Fields]) AND ("skin neoplasms"[MeSH Terms] OR ("skin"[All Fields] AND "neoplasms"[All Fields]) OR "skin neoplasms"[All Fields] OR ("skin"[All Fields] AND "cancer"[All Fields]) OR "skin cancer"[All Fields])	None
PubMed	nanoparticle photodynamic therapy melanoma	("nanoparticle s"[All Fields] OR "nanoparticles"[MeSH Terms] OR "nanoparticles"[All Fields] OR "nanoparticle"[All Fields]) AND ("photochemotherapy"[MeSH Terms] OR "photochemotherapy"[All Fields] OR ("photodynamic"[All Fields] AND "therapy"[All Fields]) OR "photodynamic therapy"[All Fields]) AND ("melanoma"[MeSH Terms] OR "melanoma"[All Fields] OR "melanomas"[All Fields] OR "melanoma s"[All Fields])	None
PubMed	nanoparticle photodynamic therapy cutaneous squamous cell carcinoma	("nanoparticle s"[All Fields] OR "nanoparticles"[MeSH Terms] OR "nanoparticles"[All Fields] OR "nanoparticle"[All Fields]) AND ("photochemotherapy"[MeSH Terms] OR "photochemotherapy"[All Fields] OR ("photodynamic"[All Fields] AND "therapy"[All Fields]) OR "photodynamic therapy"[All Fields]) AND ("cutaneous squamous cell carcinoma"[MeSH Terms] OR ("cutaneous"[All Fields] AND "squamous"[All Fields] AND "cell"[All Fields] AND "carcinoma"[All Fields]) OR "cutaneous squamous cell carcinoma"[All Fields])	None

PubMed	nanoparticle photodynamic therapy basal cell carcinoma	("nanoparticle s"[All Fields] OR "nanoparticles"[MeSH Terms] OR "nanoparticles"[All Fields] OR "nanoparticle"[All Fields]) AND ("photochemotherapy"[MeSH Terms] OR "photochemotherapy"[All Fields] OR ("photodynamic"[All Fields] AND "therapy"[All Fields]) OR "photodynamic therapy"[All Fields]) AND ("basal cell carcinoma"[MeSH Terms] OR ("basal"[All Fields] AND "cell"[All Fields] AND "carcinoma"[All Fields]) OR "basal cell carcinoma"[All Fields])	None
PubMed	nanoemulsion photodynamic therapy actinic keratosis	("nanoemulsion"[All Fields] OR "nanoemulsions"[All Fields] OR "nanoemulsive"[All Fields]) AND ("photochemotherapy"[MeSH Terms] OR "photochemotherapy"[All Fields] OR ("photodynamic"[All Fields] AND "therapy"[All Fields]) OR "photodynamic therapy"[All Fields]) AND ("keratosis, actinic"[MeSH Terms] OR ("keratosis"[All Fields] AND "actinic"[All Fields]) OR "actinic keratosis"[All Fields] OR ("actinic"[All Fields] AND "keratosis"[All Fields]))	None
PubMed	photodynamic therapy immunogenic cell death melanoma	("photochemotherapy"[MeSH Terms] OR "photochemotherapy"[All Fields] OR ("photodynamic"[All Fields] AND "therapy"[All Fields]) OR "photodynamic therapy"[All Fields]) AND ("immunogenic cell death"[MeSH Terms] OR ("immunogenic"[All Fields] AND "cell"[All Fields] AND "death"[All Fields]) OR "immunogenic cell death"[All Fields]) AND ("melanoma"[MeSH Terms] OR "melanoma"[All Fields] OR "melanomas"[All Fields] OR "melanoma s"[All Fields])	None
PubMed	photoimmunotherapy nanoparticles melanoma	"photoimmunotherapy"[All Fields] AND ("nanoparticle s"[All Fields] OR "nanoparticles"[MeSH Terms] OR "nanoparticles"[All Fields] OR "nanoparticle"[All Fields]) AND ("melanoma"[MeSH Terms] OR "melanoma"[All Fields] OR "melanomas"[All Fields] OR "melanoma s"[All Fields])	None
PubMed	photodynamic therapy hypoxia nanoparticles melanoma	("photochemotherapy"[MeSH Terms] OR "photochemotherapy"[All Fields] OR ("photodynamic"[All Fields] AND	None

		"therapy"[All Fields]) OR "photodynamic therapy"[All Fields]) AND ("hypoxia"[MeSH Terms] OR "hypoxia"[All Fields] OR "hypoxia s"[All Fields] OR "hypoxias"[All Fields]) AND ("nanoparticle s"[All Fields] OR "nanoparticles"[MeSH Terms] OR "nanoparticles"[All Fields] OR "nanoparticle"[All Fields]) AND ("melanoma"[MeSH Terms] OR "melanoma"[All Fields] OR "melanomas"[All Fields] OR "melanoma s"[All Fields])	
PubMed	photodynamic therapy microneedles skin cancer	("photochemotherapy"[MeSH Terms] OR "photochemotherapy"[All Fields] OR ("photodynamic"[All Fields] AND "therapy"[All Fields]) OR "photodynamic therapy"[All Fields]) AND ("microneedle drug delivery"[MeSH Terms] OR ("microneedle"[All Fields] AND "drug"[All Fields] AND "delivery"[All Fields]) OR "microneedle drug delivery"[All Fields] OR "microneedle"[All Fields] OR "microneedles"[All Fields] OR "percutaneous collagen induction"[MeSH Terms] OR ("percutaneous"[All Fields] AND "collagen"[All Fields] AND "induction"[All Fields]) OR "percutaneous collagen induction"[All Fields] OR "microneedling"[All Fields]) AND ("skin neoplasms"[MeSH Terms] OR ("skin"[All Fields] AND "neoplasms"[All Fields]) OR "skin neoplasms"[All Fields] OR ("skin"[All Fields] AND "cancer"[All Fields]) OR "skin cancer"[All Fields])	None
PubMed	organelle targeted photodynamic therapy melanoma nanoparticles	("organell"[All Fields] OR "organelle s"[All Fields] OR "organelles"[MeSH Terms] OR "organelles"[All Fields] OR "organelle"[All Fields] OR "organells"[All Fields]) AND ("target"[All Fields] OR "targetability"[All Fields] OR "targetable"[All Fields] OR "targeted"[All Fields] OR "targeting"[All Fields] OR "targetings"[All Fields] OR "targets"[All Fields] OR "targetted"[All Fields] OR "targetting"[All Fields]) AND ("photochemotherapy"[MeSH Terms] OR "photochemotherapy"[All Fields] OR ("photodynamic"[All Fields] AND "therapy"[All Fields]) OR "photodynamic therapy"[All Fields]) AND ("melanoma"[MeSH Terms] OR "melanoma"[All Fields] OR	None

		"melanomas"[All Fields] OR "melanoma s"[All Fields]) AND ("nanoparticle s"[All Fields] OR "nanoparticles"[MeSH Terms] OR "nanoparticles"[All Fields] OR "nanoparticle"[All Fields])	
PubMed	theranostic nanoparticles photodynamic therapy melanoma	("precision medicine"[MeSH Terms] OR ("precision"[All Fields] AND "medicine"[All Fields]) OR "precision medicine"[All Fields] OR "theranostic"[All Fields] OR "theranostics"[All Fields]) AND ("nanoparticle s"[All Fields] OR "nanoparticles"[MeSH Terms] OR "nanoparticles"[All Fields] OR "nanoparticle"[All Fields]) AND ("photochemotherapy"[MeSH Terms] OR "photochemotherapy"[All Fields] OR ("photodynamic"[All Fields] AND "therapy"[All Fields]) OR "photodynamic therapy"[All Fields]) AND ("melanoma"[MeSH Terms] OR "melanoma"[All Fields] OR "melanomas"[All Fields] OR "melanoma s"[All Fields])	None
PubMed	photodynamic therapy tumor microenvironment melanoma nanoparticles	("photochemotherapy"[MeSH Terms] OR "photochemotherapy"[All Fields] OR ("photodynamic"[All Fields] AND "therapy"[All Fields]) OR "photodynamic therapy"[All Fields]) AND ("tumour microenvironment"[All Fields] OR "tumor microenvironment"[MeSH Terms] OR ("tumor"[All Fields] AND "microenvironment"[All Fields]) OR "tumor microenvironment"[All Fields]) AND ("melanoma"[MeSH Terms] OR "melanoma"[All Fields] OR "melanomas"[All Fields] OR "melanoma s"[All Fields]) AND ("nanoparticle s"[All Fields] OR "nanoparticles"[MeSH Terms] OR "nanoparticles"[All Fields] OR "nanoparticle"[All Fields])	None
PMC	nanomedicine photodynamic therapy skin cancer	("nanomedicinal"[All Fields] OR "nanomedicine"[MeSH Terms] OR "nanomedicine"[All Fields] OR "nanomedicines"[All Fields]) AND ("photochemotherapy"[MeSH Terms] OR "photochemotherapy"[All Fields] OR ("photodynamic"[All Fields] AND "therapy"[All Fields]) OR "photodynamic therapy"[All Fields]) AND ("skin neoplasms"[MeSH Terms] OR ("skin"[All Fields] AND "neoplasms"[All Fields]) OR	None

		"skin neoplasms"[All Fields] OR ("skin"[All Fields] AND "cancer"[All Fields]) OR "skin cancer"[All Fields])	
PMC	nanoparticle photodynamic therapy melanoma	("nanoparticle s"[All Fields] OR "nanoparticles"[MeSH Terms] OR "nanoparticles"[All Fields] OR "nanoparticle"[All Fields]) AND ("photochemotherapy"[MeSH Terms] OR "photochemotherapy"[All Fields] OR ("photodynamic"[All Fields] AND "therapy"[All Fields]) OR "photodynamic therapy"[All Fields]) AND ("melanoma"[MeSH Terms] OR "melanoma"[All Fields] OR "melanomas"[All Fields] OR "melanoma s"[All Fields])	None
PMC	nanoparticle photodynamic therapy cutaneous squamous cell carcinoma	("nanoparticle s"[All Fields] OR "nanoparticles"[MeSH Terms] OR "nanoparticles"[All Fields] OR "nanoparticle"[All Fields]) AND ("photochemotherapy"[MeSH Terms] OR "photochemotherapy"[All Fields] OR ("photodynamic"[All Fields] AND "therapy"[All Fields]) OR "photodynamic therapy"[All Fields]) AND ("cutaneous squamous cell carcinoma"[MeSH Terms] OR ("cutaneous"[All Fields] AND "squamous"[All Fields] AND "cell"[All Fields] AND "carcinoma"[All Fields]) OR "cutaneous squamous cell carcinoma"[All Fields])	None
PMC	nanoparticle photodynamic therapy basal cell carcinoma	("nanoparticle s"[All Fields] OR "nanoparticles"[MeSH Terms] OR "nanoparticles"[All Fields] OR "nanoparticle"[All Fields]) AND ("photochemotherapy"[MeSH Terms] OR "photochemotherapy"[All Fields] OR ("photodynamic"[All Fields] AND "therapy"[All Fields]) OR "photodynamic therapy"[All Fields]) AND ("basal cell carcinoma"[MeSH Terms] OR ("basal"[All Fields] AND "cell"[All Fields] AND "carcinoma"[All Fields]) OR "basal cell carcinoma"[All Fields])	None
PMC	nanoemulsion photodynamic therapy actinic keratosis	("nanoemulsion"[All Fields] OR "nanoemulsions"[All Fields] OR "nanoemulsive"[All Fields]) AND ("photochemotherapy"[MeSH Terms] OR "photochemotherapy"[All Fields] OR ("photodynamic"[All Fields] AND "therapy"[All Fields]) OR "photodynamic	None

		therapy"[All Fields]) AND ("keratosis actinic"[MeSH Terms] OR ("keratosis"[All Fields] AND "actinic"[All Fields]) OR "actinic keratosis"[All Fields] OR ("actinic"[All Fields] AND "keratosis"[All Fields]))	
PMC	photodynamic therapy immunogenic cell death melanoma	("photochemotherapy"[MeSH Terms] OR "photochemotherapy"[All Fields] OR ("photodynamic"[All Fields] AND "therapy"[All Fields]) OR "photodynamic therapy"[All Fields]) AND ("immunogenic cell death"[MeSH Terms] OR ("immunogenic"[All Fields] AND "cell"[All Fields] AND "death"[All Fields]) OR "immunogenic cell death"[All Fields]) AND ("melanoma"[MeSH Terms] OR "melanoma"[All Fields] OR "melanomas"[All Fields] OR "melanoma s"[All Fields])	None
PMC	photoimmunotherapy nanoparticles melanoma	"photoimmunotherapy"[All Fields] AND ("nanoparticle s"[All Fields] OR "nanoparticles"[MeSH Terms] OR "nanoparticles"[All Fields] OR "nanoparticle"[All Fields]) AND ("melanoma"[MeSH Terms] OR "melanoma"[All Fields] OR "melanomas"[All Fields] OR "melanoma s"[All Fields])	None
PMC	photodynamic therapy hypoxia nanoparticles melanoma	("photochemotherapy"[MeSH Terms] OR "photochemotherapy"[All Fields] OR ("photodynamic"[All Fields] AND "therapy"[All Fields]) OR "photodynamic therapy"[All Fields]) AND ("hypoxia"[MeSH Terms] OR "hypoxia"[All Fields] OR "hypoxia s"[All Fields] OR "hypoxias"[All Fields]) AND ("nanoparticle s"[All Fields] OR "nanoparticles"[MeSH Terms] OR "nanoparticles"[All Fields] OR "nanoparticle"[All Fields]) AND ("melanoma"[MeSH Terms] OR "melanoma"[All Fields] OR "melanomas"[All Fields] OR "melanoma s"[All Fields])	None
PMC	photodynamic therapy microneedles skin cancer	("photochemotherapy"[MeSH Terms] OR "photochemotherapy"[All Fields] OR ("photodynamic"[All Fields] AND "therapy"[All Fields]) OR "photodynamic therapy"[All Fields]) AND ("microneedle drug delivery"[MeSH Terms] OR ("microneedle"[All Fields] AND "drug"[All Fields] AND "delivery"[All Fields]) OR	None

		"microneedle drug delivery"[All Fields] OR "microneedle"[All Fields] OR "microneedles"[All Fields] OR "percutaneous collagen induction"[MeSH Terms] OR ("percutaneous"[All Fields] AND "collagen"[All Fields] AND "induction"[All Fields]) OR "percutaneous collagen induction"[All Fields] OR "microneedling"[All Fields]) AND ("skin neoplasms"[MeSH Terms] OR ("skin"[All Fields] AND "neoplasms"[All Fields]) OR "skin neoplasms"[All Fields] OR ("skin"[All Fields] AND "cancer"[All Fields]) OR "skin cancer"[All Fields])	
PMC	organelle targeted photodynamic therapy melanoma nanoparticles	("organell"[All Fields] OR "organelle s"[All Fields] OR "organelles"[MeSH Terms] OR "organelles"[All Fields] OR "organelle"[All Fields] OR "organells"[All Fields]) AND ("target"[All Fields] OR "targetability"[All Fields] OR "targetable"[All Fields] OR "targeted"[All Fields] OR "targeting"[All Fields] OR "targetings"[All Fields] OR "targets"[All Fields] OR "targetted"[All Fields] OR "targetting"[All Fields]) AND ("photochemotherapy"[MeSH Terms] OR "photochemotherapy"[All Fields] OR ("photodynamic"[All Fields] AND "therapy"[All Fields]) OR "photodynamic therapy"[All Fields]) AND ("melanoma"[MeSH Terms] OR "melanoma"[All Fields] OR "melanomas"[All Fields] OR "melanoma s"[All Fields]) AND ("nanoparticle s"[All Fields] OR "nanoparticles"[MeSH Terms] OR "nanoparticles"[All Fields] OR "nanoparticle"[All Fields])	None
PMC	theranostic nanoparticles photodynamic therapy melanoma	("precision medicine"[MeSH Terms] OR ("precision"[All Fields] AND "medicine"[All Fields]) OR "precision medicine"[All Fields] OR "theranostic"[All Fields] OR "theranostics"[All Fields]) AND ("nanoparticle s"[All Fields] OR "nanoparticles"[MeSH Terms] OR "nanoparticles"[All Fields] OR "nanoparticle"[All Fields]) AND ("photochemotherapy"[MeSH Terms] OR "photochemotherapy"[All Fields] OR ("photodynamic"[All Fields] AND "therapy"[All Fields]) OR "photodynamic therapy"[All Fields]) AND ("melanoma"[MeSH Terms] OR	None

		"melanoma"[All Fields] OR "melanomas"[All Fields] OR "melanoma s"[All Fields])	
PMC	photodynamic therapy tumor microenvironment melanoma nanoparticles	("photochemotherapy"[MeSH Terms] OR "photochemotherapy"[All Fields] OR ("photodynamic"[All Fields] AND "therapy"[All Fields]) OR "photodynamic therapy"[All Fields]) AND ("tumour microenvironment"[All Fields] OR "tumor microenvironment"[MeSH Terms] OR ("tumor"[All Fields] AND "microenvironment"[All Fields]) OR "tumor microenvironment"[All Fields]) AND ("melanoma"[MeSH Terms] OR "melanoma"[All Fields] OR "melanomas"[All Fields] OR "melanoma s"[All Fields]) AND ("nanoparticle s"[All Fields] OR "nanoparticles"[MeSH Terms] OR "nanoparticles"[All Fields] OR "nanoparticle"[All Fields])	None
